# Polyhydroxyalkanoates production from short and medium chain carboxylic acids by *Paracoccus homiensis*

**DOI:** 10.1038/s41598-022-11114-x

**Published:** 2022-05-04

**Authors:** Karolina Szacherska, Krzysztof Moraczewski, Piotr Rytlewski, Sylwester Czaplicki, Sławomir Ciesielski, Piotr Oleskowicz-Popiel, Justyna Mozejko-Ciesielska

**Affiliations:** 1 Department of Microbiology and Mycology, Faculty of Biology and Biotechnology, University of Warmia and Mazury in Olsztyn, 10-719 Olsztyn, Poland; 2 Institute of Materials Engineering, Kazimierz Wielki University, 85-064 Bydgoszcz, Poland; 3 Department of Plant Food Chemistry and Processing, University of Warmia and Mazury in Olsztyn, Pl. Cieszyński 1, 10-726 Olsztyn, Poland; 4 Department of Environmental Biotechnology, University of Warmia and Mazury in Olsztyn, 10-719 Olsztyn, Poland; 5 Water Supply and Bioeconomy Division, Faculty of Environmental Engineering and Energy, Poznan University of Technology, 60-965 Poznan, Poland

**Keywords:** Environmental biotechnology, Applied microbiology, Environmental microbiology

## Abstract

The aim of this study was to evaluate an effect of short and medium chain carboxylic acids (CAs) rich stream derived from acidogenic mixed culture fermentation of acid whey on polyhydroxyalkanoates (PHAs) synthesis by *Paracoccus homiensis* and compare it with the impact of individual synthetic CAs. The obtained results confirmed that the analyzed bacterium is able to metabolize synthetic CAs as the only carbon sources in the growth medium with maximum PHAs production yields of 26% of cell dry mass (CDM). The replacement of the individual CAs by a CAs-rich residual stream was found to be beneficial for the *Paracoccus homiensis* growth. The highest biomass concentration reached about 2.5 g/L with PHAs content of 17% of CDM. The purified PHAs were identified as poly(3-hydroxybutyrate-co-3-hydroxyvalerate) by applying gas chromatography coupled with mass spectrometry, Fourier transform infrared spectroscopic spectra and UV–Vis spectra. Furthermore, a differential scanning calorimetric, thermogravimetric and water contact angle analysis proved that the extracted copolymers have useful properties. The obtained data are promising in the perspective of developing a microbial PHAs production as a part of an integrated valorization process of high CAs content waste-derived streams.

## Introduction

Due to the dependence of synthetic polymers on fossil fuels and the sustainability issues related to plastic waste, biopolymers have attracted much attention in the last few decades^1^. Polyhydroxyalkanoates (PHAs) are especially attractive because being biodegradable, non-toxic and biocompatible they can replace conventional petroleum-based polymers in the near future^2^. Furthermore, they are produced in biotechnological processes, which enables to control their chemical structure and physico-thermal properties during cultivations of microorganisms^3^. PHAs are accumulated in bacterial cells as carbon and energy reserves under environmental stress in the form of granules with a diameter of 0.2–1.0 mm^4^. They are classified according to the number of carbon atoms presents in the biopolymer molecule. There are short-chain-length PHA (scl-PHA) containing 3–5 carbon atoms in the molecule, medium-chain-length PHA (mcl-PHA) which contains 6–14 carbon atoms in the molecule, and long-chain-length PHA (lcl-PHA) with more than 15 carbon atoms^5^. Moreover, because of their unique properties they have great potential in biomedical, agricultural and industrial applications^6^. However, their production on the commercial scale is still limited due to their high production costs compared to their synthetic alternatives. Thus, there is a need for the optimization of novel fermentation processes using renewable, low value carbon sources. Furthermore, to improve physicochemical properties of biopolymers, there is a need to study the biosynthesis process in the context of the possibility to synthesize not only poly(3-hydroxybutyrate) homopolymer [P(3HB)] but also co-polymers with different content of the monomers. So far, various low value substrates have been studied as potential carbon sources for PHAs production, but most of them required additional pretreatment steps that further increased the PHA production costs^7,8^.

A promising substrate for PHAs production are short and medium chain carboxylic acids (CAs) generated by acidogenic anaerobic mixed culture fermentation (MCF). Significant research attention is being given to CAs since they can be converted into high-value bioproducts such as PHAs^9^. The utilization of CAs for PHAs synthesis will not only make cost-effective PHAs production feasible but also will be beneficial for the environment since CAs are produced through degradation of organic wastes largely available worldwide. The synthesis of PHAs from synthetic CAs has been conducted by employing bacteria such as: *Pseudomonas* sp. ST2^10^; *Ralstonia eutropha*^11^ or *Haloferax mediterranei*^12^.

To the best of our knowledge, only in a few publications the potential of microorganisms to convert CAs-rich streams from MCF using organic wastes into value-added PHAs was evaluated^13–16^. None of this study evaluated in details properties of the extracted biopolyesters. Moreover, there is still a lack of studies that consider the biopolyesters production from CAs using species belong to *Paracoccus* genera, even though these bacteria have substantial biotechnological potential^17^. Hence, in this study, the individual synthetic CAs and those derived from MCF of acid whey were used as feedstocks to determine the possibility of producing PHAs by *Paracoccus homiensis.* Additionally, the impact of the type of CAs and CAs-rich stream concentration on the content of biopolymers in bacterial cells, the growth of the bacterial producer and the monomeric composition of the final bioproducts were determined. Furthermore, the extracted PHAs were comprehensively characterized taking into account their physico-thermal and water related properties.

## Results and discussion

### Growth and PHAs production using individual synthetic CAs

To determine a type and a suitable concentration of individual CAs that guarantee the effective growth of the *Paracoccus homiensis* and efficient PHAs production, the culture medium was supplemented with the different concentrations of acetic acid, propionic acid, butyric acid, valeric acid and caproic acid. The same growth medium conditions and the amount of inoculum were maintained in all cultivations.

**Figure 1:**
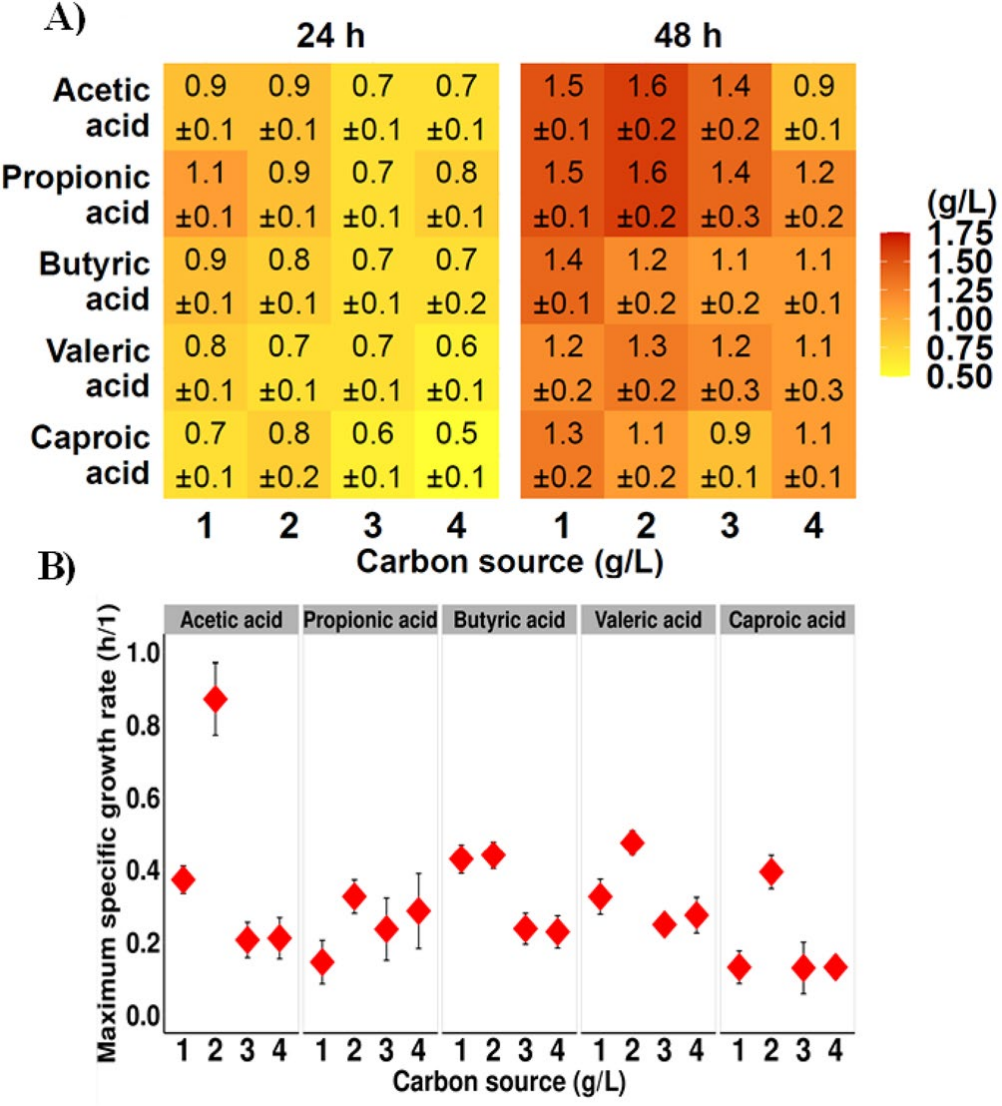
Profiles of *P. homiensis* growth in the medium supplemented with individual short and medium chain carboxylic acids as the only carbon source. (**A**) Biomass concentration (g/L) in 24 h and 48 h of the cultivations; (**B**) maximum specific growth rate (μ_max_).

As shown in Fig. 1A,B all tested CAs were utilized to support cell growth and PHAs synthesis and accumulation. During all cultivations, the growth rate depended on the individual CAs concentration. In all experiments, the maximum specific growth rate (μ_max_) reached a higher value than 0.1/h (Fig. 1B). Using 2 g/L of acetic acid, both maximum μ_max_ (0.87/h) and biomass concentration (1.6 g/L) were obtained. It was previously suggested that acetic acid is less toxic and could be easier metabolized by bacteria than other tested CAs^18^. Saraphirom et al.^19^ revealed that when acetic acid is present in the medium microorganisms prefer to utilize acetic acid than butyric acid. Our study showed that the addition of 2 g/L of propionic acid or valeric acid also resulted in higher biomass concentration compared to other analyzed concentrations of these CAs. For butyric and caproic acids the most preferable concentration to support the bacterial growth was 1 g/L, at this level the CDM values reached 1.4 and 1.3 g/L, respectively. Moreover, the results confirmed that with an increase of all CAs concentration to 4 g/L, μ_max_ and CDM value decreased. However, the CDM concentration was higher at 48 h of all treatments compared with 24 h. The reported differences in the μ_max_ values were related to different metabolic pathways that were involved in the utilization of CAs. Acetic acid, present in the culture medium can be initially converted to acetyl-CoA which is one of an intermediate in PHA synthesis. Then, two acetyl-CoA moieties are transformed to acetoacetyl-CoA which is needed for the synthesis of 3HB monomer. This monomer could be directly synthesized via acetoacetyl-CoA when butyric acid is present in the growth medium. For propionic acid the carbonyl carbon of propionyl-CoA is eliminated that enable to synthesize both 3HB and 3HV units^20^.

**Figure 2:**
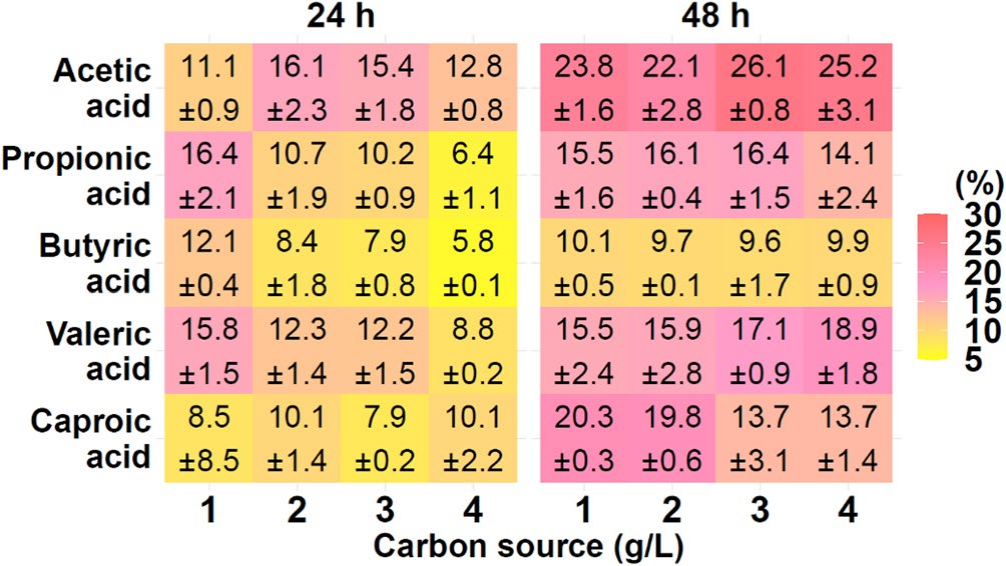
PHAs content (% of CDM) in *P. homiensis* cells in 24 h and 48 h during growth in the medium supplemented with individual short and medium chain carboxylic acids as the only carbon source.

Our results indicated that the PHAs content in *P. homiensis* cells was dependent on the type and concentration of individual CAs. The analyzed bacteria were able to produce PHAs in all conducted cultivations with CAs. As expected, no PHAs were synthesized in bacterial cells grown on Bacto Marine Broth (treated as a control). As may be observed from data reported in Fig. 2 the highest PHAs production was obtained when acetic acid was added as a single carbon source. However, the statistically significant difference (*P* = 0.014) was found only between cultivations supplemented with acetic and butyric acid. The best accumulation of the PHAs from this CA was achieved at 3 g/L (26.1% of CDM). Also, this concentration was ideal for PHA production by *P. homiensis* grown on propionic acid, however, about 1.6 fold lower PHA accumulation value was observed compared with acetic acid. The bacteria could efficiently metabolized valeric acid at 4 g/L to produce higher content of the biopolymer (18.9% of CDM) compared to other concentrations of this substrate. PHA synthesis and accumulation by *P. homiensis* was inhibited as the caproic acid concentration increased reaching 20.3% and 13.7% of CDM at 1 g/L and 4 g/L, respectively. This could be due to inhibitory effect of the substrate for the accumulation mechanisms of the analyzed bacterial cells. Too high CAs level could have reduced the proton gradient across the cell membrane and increased osmotic pressure of the cells due to their need to pump out anions to survive under acidic pH conditions in the growth medium. Moreover, the presence of organic acids in cytoplasm results in releasing protons that caused its acidification and more energy is used to maintain the gradient inside the cell^14^. Thus, higher CAs concentration used in our study may have resulted in low acid utilization and PHA concentration. Therefore, the optimization conducted within our studies seems to be essential as the undissociated CAs molecules could enter the cytoplasm and in a consequence could be activated and utilized into CO_2_, bacterial cell growth or PHA synthesis only when CAs are present at appropriate levels^21,22^. Thus, CAs concentration should be carefully regulated during bacterial cultivations. In general, the lowest biopolymer content in all treatments was observed when the bacteria were cultured on butyric acid at all analyzed concentrations (from 9.7 to 10.1% PHAs of CDM). Kedia et al.^23^ have also revealed the inhibitory effect of butyric acid for PHAs storage ability by *Cupriavidus necator,* a lower PHA concentration was observed when this acid was used as the only substrate compared to acetic acid. Nevertheless, these results are not in accordance with the data reported by Chakraborty et al.^24^ for *Ralstonia eutropha* which grown on butyric acid and yielded the best results of PHA concentration compared to acetic acid. It could be suggested that for *P. homiensis* butyric acid is not energetically efficient as other CAs for biopolyesters synthesis and accumulation. Furthermore, the cell mass concentration could be the primary factor that affect this acid tolerance of the analyzed bacteria^25^. During the *P. homiensis* growth in the medium supplemented with butyric acid, the lowest CDM level was achieved.

### Growth and PHAs using CAs-rich stream

**Figure 3:**
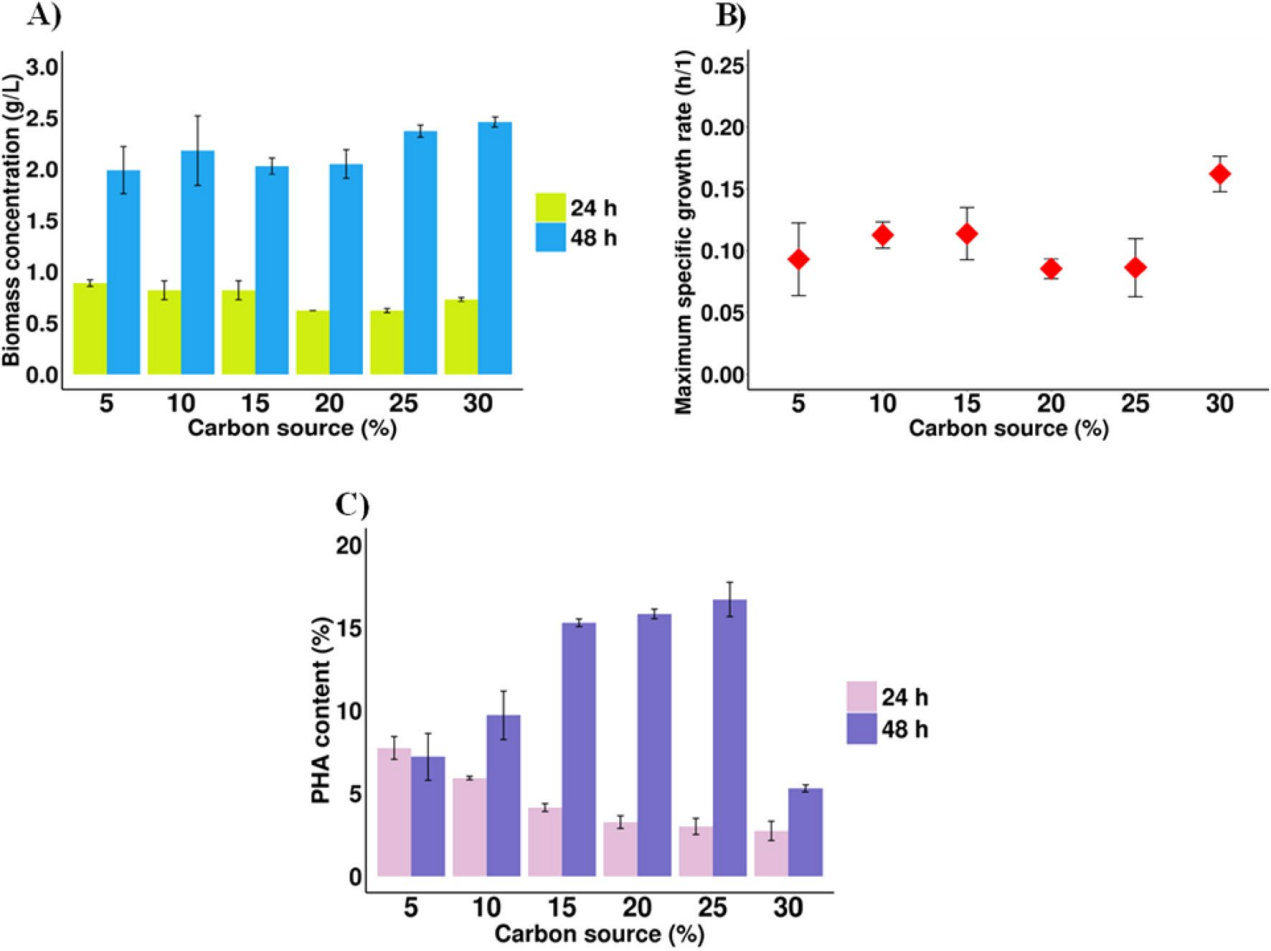
Profiles of *P. homiensis* growth and PHAs content in the medium supplemented with different concentrations of CAs-rich stream from acidogenic mixed culture fermentation of acid whey as the only carbon source. (**A**) Biomass concentration at 24 h and 48 h of the cultivations; (**B**) Maximum specific growth rate (μ_max_); (**C**) PHAs content in *P. homiensis* cells at 24 h and 48 h. Mean values are calculated from triplicate measurements.

Studies on CAs utilization for the PHA biosynthesis by *P. homiensis* have not been described in literature so far. The production of these biopolyesters by other well-known microorganisms are limited to the use of individual synthetic CAs. Therefore, to determine the effect of CAs derived from acid whey in more detail, CAs-rich stream was added to the Bacto Marine Broth at varying concentrations (from 5 to 30% v/v). During all cultivations, an increase in the absorbance of the cultures was observed. The highest μ_max_ (0.16/h) and CDM (2.5 g/L) value was determined when 30% of CAs-rich stream was added to the culture broth (Fig. 3A,B). PHA production was observed under all CAs-rich stream concentrations. At 24 h, the PHAs content decreased when the carbon source concentration increased (from 7.7 to 2.7% of CDM). However, at the end of the experiments, the PHAs content was the highest in *P. homiensis* cells cultivated in culture medium containing 25% CAs-rich stream, about 16.7% of CDM. As may be observed in Fig. 3C, the further increase of the concentration of this waste carbon source resulted in a decrease of PHAs content. PHAs were detected in a trace amount in the bacterial cells cultured in the medium supplemented with 30% CAs-rich stream (5.3% of CDM). Relatively lower PHA content from CAs-rich stream compared to the individual CAs proved that CAs-rich stream had more inhibitory effect due to its complexity. The inhibition of PHA biosynthesis could have been caused by the CAs-rich stream which may interfered with the proton gradient mechanism^15^. Furthermore, quite low PHA productivity could be explained by the presence of butyric acid as the dominant component in the CAs-rich stream which as a single carboxylic acid in the medium resulted in the lowest PHAs production efficiency compared with others. It should be also noted that the presence of high CAs concentration can reduce growth rate, therefore most researchers add such carbon sources after reaching by bacteria its maximum growth, whereas in our study CAs-rich stream was added at the beginning of the cultivations. Furthermore, the experiments were conducted in 250-mL shake flasks in which a control of the parameters like aeration rate, pH value is not possible. It could be presumed that the above mentioned factors might have affected achievement of higher PHA concentration. However, the obtained PHA content was about twofold higher than that reported by Vu et al.^16^ who cultivated *Bacillus megaterium* also on the CAs-rich stream from the acidogenic fermentation of food waste. To improve the PHA yields, CAs feeding adjustment, optimization of oxygen, pH control is needed.

### Characterization of extracted PHA copolymers

#### PHA biopolymer repeat-units composition

The extracted PHAs from all performed cultivations fed with individual CAs and CAs-rich stream were subjected to gas chromatography analysis to provide information about their repeat-units composition (Table 1). Our data indicate that the monomeric composition varied with the carbon sources concentration and the monomers content was changeable at different process time.

**Table 1:** Monomeric composition of PHAs produced by *P. homiensis* grown on synthetic and waste derived short and medium chain carboxylic acids.

Carbon source	Substrate concentration (g/L; %*)	PHA monomeric composition (mol%)			
		24 h		48 h	
		3HB	3HV	3HB	3HV
Acetic acid	1	99.2	0.8	98.8	1.2
	2	99.4	0.6	98.8	1.2
	3	99.3	0.7	98.7	1.3
	4	99.5	0.5	99.1	0.9
Propionic acid	1	93.2	6.8	91.2	8.8
	2	100.0	0.0	92.7	7.3
	3	94.3	5.7	90.9	9.1
	4	93.6	6.4	91.3	8.7
Butyric acid	1	99.2	0.8	99.0	1.0
	2	99.3	0.7	99.1	0.9
	3	99.4	0.6	99.0	1.0
	4	100.0	0.0	99.3	0.7
Valeric acid	1	40.1	59.9	37.6	62.4
	2	17.5	82.5	33.9	66.1
	3	18.2	81.8	16.6	83.4
	4	14.7	85.3	13.0	87.0
Caproic acid	1	98.9	1.1	98.1	1.9
	2	99.0	1.0	98.8	1.2
	3	98.8	1.2	98.5	1.5
	4	98.9	1.1	98.6	1.4
CAs—rich stream	5*	98.6	1.4	98.5	1.5
	10*	97.6	2.4	98.8	1.2
	15*	97.3	2.7	98.3	1.7
	20*	92.5	7.5	98.5	1.5
	25*	95.9	4.1	98.4	1.6
	30*	95.1	4.9	98.1	1.9

3HB, 3-hydroxybutyrate; 3HV, 3-hydroxyvalerate; CAs, carboxylic acids.

%* - an unit of the concentration of CAs-rich stream

The obtained results proved that *P. homiensis* cultured on acetic acid and caproic acid showed a tendency to synthesize the biopolymer consisted of high 3HB monomer and lower 3HV monomer for all concentrations and both time intervals. The 3HV content increased with prolonging the process time. The data revealed that the *P. homiensis* fed at 2 g/L of propionic acid and 4 g/L of butyric acid produced P(3HB) homopolymer. At other concentrations of those carboxylates the 3HB and 3HV monomers were detected. However, higher 3HV content was observed in the cultivations with propionic acid at both 24 h and 48 h of the processes. The highest 3HV content was confirmed in the P(3HB-co-3HV) copolymer extracted from the cells grown on valeric acid. The 3HV content was higher than 3HB content at both 24 and 48 h and this fraction showed higher share with increase in valeric acid concentration, from 59.9 to 85.3 mol% at 24 h and from 62.4 to 87.0 mol% at 48 h.

Our data showed that the utilization of CAs-rich stream led to the synthesis and accumulation of the P(3HB-co-3HV) copolymer. Due to the presence in the CAs-rich stream high amounts of acetic acid and butyric acid, low concentration of valeric acid, and the absence of propionic acid the extracted copolymer consisted of the lower 3HV content than 3HB. Furthermore, the GC results proved that the 3HV content was higher at 24 h compared to 48 h (*P* = 0.045). At 24 h of cultivation, the 3HV content was positively correlated with the CAs-rich stream concentrations (Spearman’s correlation coefficient = 0.83). Surprisingly, the highest 3HV content (7.5 mol%) was observed when 20% of CAs-rich stream was added. At 48 h correlation between the 3HV content and the CAs-stream concentrations was not observed. Generally, higher 3HV content was observed when higher CAs-stream concentrations were applied. The possibility of the copolymer production using CAs-rich stream is very attractive since pure P(3HB) has limited applicability due to similar melting and degradation temperatures^26^. However, the 3HV content in the extracted polymers was not sufficiently high, therefore to increase the level of this monomer the induction of the acidogenic fermentation to produce more CAs with an odd carbon number would be beneficial^15^.

#### FTIR spectra

In the FTIR spectra, several bands have been observed that can be assigned to functional groups characteristic for P(3HB-co-3HV) copolymer^27^. The spectra of PHAs samples extracted from the cultivations with acetic, propionic, butyric, caproic acid and CAs-rich streams were similar and only in the case of P(3HB-co-3HV) sample from valeric acid some differences were observed. FTIR spectra of the analyzed samples were recorded in the range from 4000 to 700 cm^−1^ (Supplementary materials 1). A band with a maximum of ~ 3333 cm^−1^ was observed only for the PHA sample produced by *P. homiensis* grown on valeric acid. It probably originates from terminal –OH groups present at the ends of the polymer chains. The presence of this band confirms earlier assumptions about the large number of shorter macromolecules (oligomers). The band in the range 3100–2700 cm^−1^ is associated with the presence of the –CH_3_ and –CH_2_ groups. Clearly larger peaks with a maximum of ~ 2925 and ~ 2852 cm^−1^ in this material are due to the higher content of 3HV units, which contain additional –CH_3_ and –CH_2_ groups. The single peak band with a maximum of ~ 1722 cm^−1^ is derived from C=O carbonyl groups present in macromolecules. In the spectra range from 1500 to 800 cm^−1^, which is a so-called fingerprint region, several bands with numerous peaks were recorded. Peaks ~ 1460, ~ 1379, ~ 980, ~ 900 and ~ 825 cm^−1^ are associated with the presence of the –CH_3_ and –CH_2_ groups, as well as C–C bonds. Whereas, peaks ~ 1278, ~ 1228, ~ 1180, ~ 1132, ~ 1101 and ~ 1053 cm^−1^ are associated with bonds in the C–O–C region. Unlike the other extracted biopolyesters in the produced copolymer using valeric acid, instead of the ~ 1278 and ~ 1228 cm^−1^ peaks, one high intensity peak derived from C–O–C bonds was recorded with a maximum at 1263 cm^−1^. The observed changes are related to the high crystallinity degree of this biopolymer. Peaks ~ 1278 and ~ 1228 cm^−1^ are very sensitive to the content of the crystalline phase^28^. In the amorphous materials in the 1330–1230 cm^−1^ range two peaks are observed, while for semi-crystalline materials only one^29^.

#### UV–VIs absorbance spectra

The spectra of the UV–VIs absorbance revealed three absorbance peaks attributed to the wavelength range from about 200 to about 400 nm (Supplementary Materials 1). The absorption bands structure is similar to the samples representing typical absorption bands for 3HB. The polymers, extracted from the bacterial cells cultured on acetic, propionic, butyric and caproic acid, showed high 3HB content (above 93%) and low 3HV content. Except for the P(3HB-co-3HV) sample from valeric acid, for which only very insignificant absorption bands were identified in that spectra region. This resulted most likely from the different chemical structure of this biopolymer for which the 3HB content is significantly reduced (only about 17 mol%).

#### Physico-thermal properties of extracted PHAs

**Figure 4:**
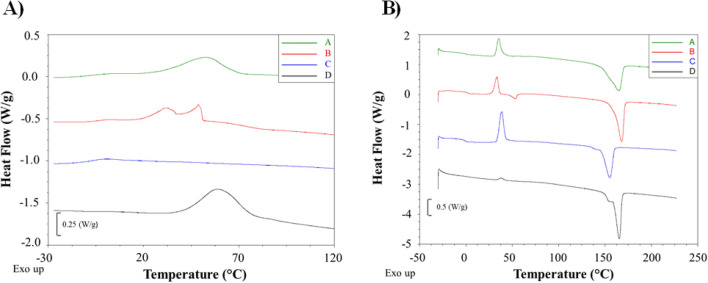
DSC curves (**A**) cooling, (**B**) second heating of selected samples: A—PHAs from acetic acid; B—PHAs from valeric acid, C—PHAs from caproic acid, D—PHAs from 15% of CAs-rich stream.

The extracted PHAs properties determine which applications these materials are best suited for and how they can be processed. Therefore, in this work, the influence of the CAs on the thermal properties of the accumulated PHAs was also analyzed (Fig. 4; Table 2; Supplementary materials 1).

**Table 2:** Thermal properties of PHAs using short and medium chain carboxylic acids as the only carbon sources.

Carbon source	PHA composition	Cooling			2nd Heating						Thermogravimetric analysis			
		T_g_ [°C]	T_c_ [°C]	ΔH_c_ [J/g]	T_g_ [°C]	T_cc_ [°C]	ΔH_cc_ [J/g]	T_m_ [°C]	ΔH_m_ [J/g]	X_c_ [%]	T_5%_ [°C]	T_50%_ [°C]	T_95%_ [°C]	T_max_ [°C]
Acetic acid	P (99.2% HB-co-0.8% HV)	− 6.6	52.9	31.2	− 0.8	35.5	47.2	165.0	78.8	29.0	232.4	258.4	296.3	262.0
Propionic acid	P (93.6% HB-co-6.4% HV)	− 3.9	nd	nd	0.8	38.0	60.2	158.9	62.5	2.1	228.7	252.6	366.6	253.0
Butyric acid	P (99.4% HB-co-0.6% HV)	− 5.6	46.6	9.0	1.9	34.2	68.9	165.8	72.1	2.9	215.9	243.2	452.3	240.2
Valeric acid	P (17.5% HB-co-82.5% HV)	− 3.1	48.9 (32.2)^a^	32.7	2.2	33.4	17.0	168.1 (53.6)^a^	72.8 (6.3)^a^	51.2	184.0	248.6	439.5	253.9
Caproic acid	P (98.8% HB-co-1.2% HV)	− 6.3	nd	nd	− 1.4	38.6	59.3	155.4	60.4	1.0	218.9	262.5	406.4	262.9
5% CAs—rich stream	P (98.5% HB-co-1.5% HV)	nd	57.2	32.7	1.5	39.7 (89.6)	31.4	165.4 (152.3)^a^	71.4	36.8	238.0	257.4	339.4	259.1
10% CAs—rich stream	P (98.8% HB-co-1.2% HV)	nd	65.4	44.5	2.0	38.2 (90.4)^b^	20.1	166.0 (155.5)^a^	66.9	42.9	234.5	251.9	527.4	253.5
15% CAs—rich stream	P (98.3% HB-co-1.7% HV)	nd	59.2	43.8	2.5	38.1 (85.4)^b^	24.6	165.4 (155.0)^a^	79.2	50.2	232.2	251.4	266.5	253.8
20% CAs—rich stream	P (98.5% HB-co-1.5% HV)	nd	59.2	51.9	2.3	38.5 (86.1)^b^	17.5	162.0 (150.3)^a^	74.9	52.7	231.2	250.2	397.2	251.9
25% CAs—rich stream	P (98.4% HB-co-1.6% HV)	nd	65.4	54.1	1.9	41.8 (86.5)^b^	21.9	163.4 (152.9)^a^	75.3	49.0	230.9	252.6	335.1	255.0
30% CAs—rich stream	P (98.1% HB-co-1.9% HV)	nd	61.1	28.9	1.0	45.0 (86.2)^b^	38.6	161.4 (149.6)^a^	65.9	25.0	221.9	247.4	409.4	247.3

nd, not detected.

^a^A distinct and separate, second crystallization and melting peak.

^b^A second cold crystallization peak.

Cooling curves, and therefore the nature of changes in the material as a result of cooling, of individual materials followed one of four types. In the first type, only the inflection related to the glass transition of the tested biomaterial was observed on the cooling curve. This type was determined for PHAs extracted from the bacteria cells grown on propionic and caproic acid. The T_g_ values determined from the cooling curves of these materials were − 3.9 and − 6.3 °C, respectively.

In the second type of cooling curve, in addition to a clear glass transition inflection, an exothermic peak associated with the crystallization of the biomaterial was also observed. This type occurred in PHAs from the cultivation supplemented with acetic and butyric acid, with the crystallization peak being much larger for the PHAs produced using acetic acid, which indicates a more intense crystallization process and more crystalline phase formation. The determined T_g_ and T_c_ values were − 6.6 and 52.9 °C for the biopolymer synthesized using acetic acid and − 5.6 and 46.6 °C using butyric acid.

A third type of cooling curve pattern was observed for the PHAs extracted from the bacterial cells grown on valeric acid. The T_g_ of this biopolymer was − 3.1 °C. Also, for this biomaterial an exothermic crystallization peak was revealed on the cooling curve. However, the crystallization peak was bimodal with clearly visible two arms of similar intensity with a maximum at 32.3 and 48.9 °C. The reason for the bimodal peak of crystallization may be the high content of 3HV mers, which significantly affected the nature of this process or the presence of a large number of macromolecules with a much lower molecular weight (oligomers). As a result, two crystalline phases with different structure and/or composition of crystallites having different crystallization temperatures may be formed during cooling.

The fourth type of cooling curves was observed for polymers obtained from CAs-rich stream. The course of the cooling process was very similar to the course of polymers obtained from acetic and butyric acid. However, the temperatures of the crystallization process and the intensity of the process were much higher. The highest crystallization temperature for this group of polymers was 65.4 °C and the enthalpy value of the crystallization process was 54.1 J/g. However, it was not possible to determine the glass transition for these samples due to the low intensity of this process.

The heating curves of the analyzed biopolymers were similar. The inflections and exothermic or endothermic peaks typical for the occurrence of glass transition, cold crystallization and melting of the crystalline phase processes were observed on all curves. T_g_ values determined from the second heating curves of individual biomaterials ranged from − 1.4 (PHAs from caproic acid) to 2.5 °C (PHAs from 15% CAs-rich stream), which agrees with the literature values that have been shown for a P(3HB-co-3HV) copolymer^30,31^.

Regardless of the nature of the crystallization during cooling, all biopolyesters showed a tendency to cold crystallization, which was the cause of the exothermic peak observed on the curves. T_cc_ values were ranged from 33.4 (PHAs from valeric acid) to 45.0 °C (PHAs from 30% CAs-rich stream). An interesting two-stage course of the cold crystallization process was observed for polymers obtained from CAs-rich stream. In the initial phase of cold crystallization there was a slight peak at about 40 °C, then the process was less intensive in a wide temperature range, which was reflected in an exothermic peak of low intensity ranging from 50 to 120 °C with a maximum around 90 °C. More significant changes were observed in the intensity of the cold crystallization process, which resulted from the course of the crystallization during cooling.

The lowest ΔH_cc_ value of 17.0 J/g was obtained for PHAs extracted from the analyzed strain grown on valeric acid, which is probably due to the different composition of this sample (high content of 3HV units) and its negative impact on susceptibility to cold crystallization. The remaining samples had significantly higher ΔH_cc_ values ranging from 47.2 (PHAs from acetic acid) to 68.9 J/g (PHAs from butyric acid). The course of the cold crystallization process depended on the course of the crystallization during cooling. The lower the intensity of the crystallization process during cooling, the greater the peak of cold crystallization, and therefore the intensity of this process.

The last phase change observed in the studied temperature range was the melting of the crystalline phase seen as an endothermic peak. The highest T_m_ value of 168.1 °C was obtained for PHAs from valeric acid, which were also characterized by a high value of ΔH_m_. This data proved the high intensity of this process and the high content of the crystalline phase. The degree of crystallinity of this sample was 51.2% and was the highest among all tested biomaterials. In addition, a second endothermic melting peak, not found in other samples, with a maximum at 53.6 °C and ΔH_m_ of 6.3 J/g was observed on the heating curve of this biopolymer. The low melting point of this peak suggests that the most likely reason for its occurrence is the melting of lower molecular weight macromolecules (oligomers), which at the same time suggests that they are the cause of the bimodal crystallization peak during cooling.

Similar melting temperatures were obtained for PHAs extracted from the cultivations supplemented with acetic acid and butyric acid. The T_m_ values of these biopolymers were 165.0 and 165.8 °C, respectively. Although the difference in ΔH_m_ was only about 7 J/g, they differed significantly in the degree of crystallinity. The calculated X_c_ values were 29.0 and 2.9%, for PHAs from acetic acid and butyric acid, respectively. These differences resulted from the crystalline phase formation stage. In the case of PHAs from butyric acid, most of the crystalline phase is formed during the cold crystallization process, so it is practically non-existent in the starting material suggesting it is amorphous. Whereas, in PHAs from acetic acid, most of the crystalline phase is formed during the cooling of this material, so the crystalline phase already occurs before it is heated, indicating that it is semi-crystalline.

The melting temperatures of the polymers obtained from CAs-rich stream ranged from 161.4 to 166 °C. However, the biggest difference compared to other polymers was the presence of a distinct second melting peak around 150 °C. The appearance of the second melting peak resulted from the two-stage process of cold crystallization, in which two crystalline phases with distinctly different melting points were formed. The degree of crystallinity of the polymers obtained from the cultivations with waste derived CAs ranged from 25.0 to 52.7%.

Lower melting temperatures and melting enthalpy changes were obtained for PHAs extracted from the cultivations with propionic and caproic acid. The T_m_ and ΔH_m_ values were 158.9 °C and 62.5 J/g, and 155.4 °C and 60.4 J/g, respectively. The intensity of the melting process of these samples was therefore similar, which with a similar course of the crystallization and the cold crystallization processes resulted in a similar degree of their crystallinity. The X_c_ values of these biopolymers were less than 3%, indicating that the starting materials had an amorphous structure.

**Figure 5:**
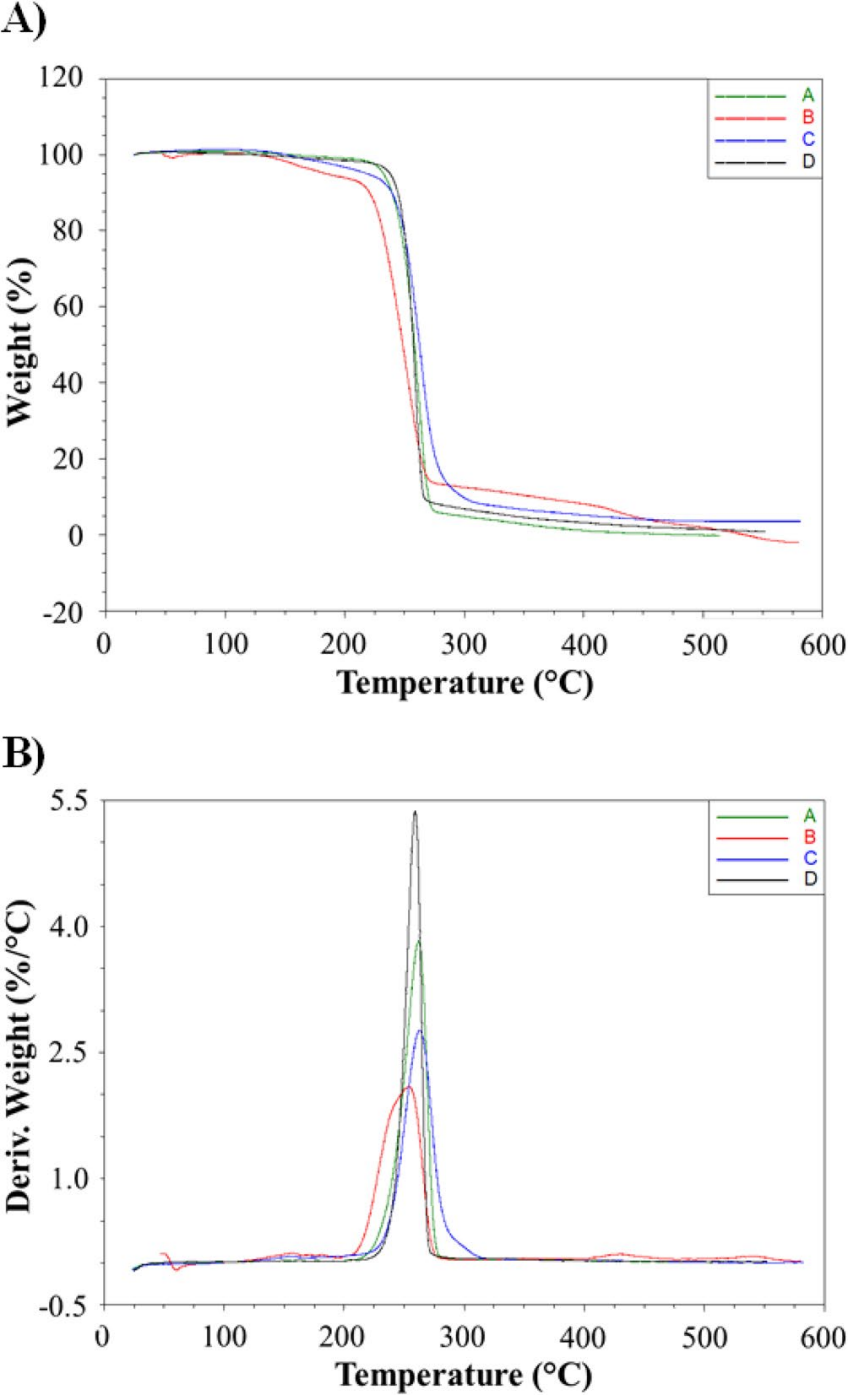
Curves (**A**) thermogravimetric and (**B**) derivative thermogravimetric of selected samples; A—PHAs from acetic acid; B—PHAs from valeric acid, C—PHAs from caproic acid; D—PHAs from 5% of CAs-rich stream.

Figure 5 shows the TG and DTG curves of selected samples (PHAs from acetic acid, valeric acid, caproic acid and 5% of CAs-rich stream, the remaining ones are shown in Supplementary materials 1). The curves of the remaining samples are similar and correspond to one of the presented, so they were not added. The numerical results of TG measurements of all samples are shown in Table 2. In the analysis of test results, as the temperature of material degradation enabling determination of its thermal resistance, a temperature of 5% loss of the mass of the tested sample was assumed (T_5%_). The T_5%_ depended on the characteristic of the weight loss process and ranged from 238.0 (PHAs from 5% CAs-rich stream) to 184.0 °C (PHAs from valeric acid).

The biopolymer extracted from the cultivation supplemented with CAs-rich stream had the highest thermal resistance among all tested biomaterials. The T_5%_ of those polymers ranged from 221.9 to 238.0 °C. The highest crystalline phase content among these tested biopolyesters, characterized by greater thermal resistance than the amorphous phase, was probably the reason for the highest thermal resistance of this materials. However, as the concentration of CAs-rich stream increased, the thermal resistance of the extracted biopolymers decreased, despite even higher degree of crystallization. T_5%_ dropped to 221.9 for PHAs obtained from 30% of CAs-rich stream.

Similar high thermal resistance was observed for the biopolymer extracted from the cultivation supplemented with acetic acid. The T_5%_ was 232.4 °C, and the highest rate of weight loss occurred at T_max_ 262.0 °C. High thermal resistance, as in the case of PHAs obtained from CAs-rich stream, resulted from the high degree of crystallinity of this material. Noteworthy is the high thermal resistance of PHAs obtained from the cultivations using propionic acid. It is similar to the resistance of PHAs extracted from the cultures with acetic acid and CAs-rich stream, despite a much lower degree of crystallinity. These polymers are characterized by a higher content of 3HV units and this may be the reason for the observed increase in thermal resistance.

The P(3HB-co-3HV) produced in the bacterial cultivations with butyric acid and caproic acid had lower thermal resistance (T_5%_ values were 215.9 and 218.9 °C, respectively) suggesting that the onset of thermal degradation of these materials was similar. However, these biopolymers differed significantly in the rate of degradation process. The T_max_ value of PHAs from caproic acid was 253.9 °C, while the value of PHAs from butyric acid was lower by more than 20 °C and was the smallest among all tested biomaterials indicating that it has the lowest thermal stability. Low T_5%_ of the above mentioned copolymers were due to the characteristic of the weight loss process. Based on the TG curves, it can be seen that the degradation is a two-step process. In the first step, a slight decrease of about 5% of the sample mass were observed, followed by a second step with rapid mass loss. The cause of the initial slight weight loss may be small-molecule compounds characterized by lower degradation temperatures contained in the biomaterial, which are residues after the synthesis process or macromolecules with shorter polymer chains. For the PHAs from valeric acid a loss of 5% of sample weight occurred already at 184.0 °C because of a high content of smaller macromolecules (oligomers). It was also confirmed by DSC analysis as well as observed evident two-step characteristic of weight loss, which in the first stage was as much as 8%. However, further rate of the mass change was similar to the other samples, which is proved by the T_m_ value of 253.9 °C.

#### Water related properties of extracted PHAs

The obtained water contact angles and calculated value of surface energy (SE) of the extracted biopolymers were summarized in Table 3. For the PHA-copolymer from propionic acid water droplet was rapidly dispersed on the surface, thus the results were not presented. As shown in Table 3 the smallest water contact angles was detected for the PHAs extracted from the *P. homiensis* cells cultured on acetic acid. The average contact angle of 29.5° suggested that this sample had strong hydrophilic properties and a high surface energy value. Whereas, the highest average water contact angle was observed for the PHAs from butyric acid indicating their high hydrophobic properties.

**Table 3:** Average water contact angles and surface energy of the analyzed PHAs films.

Carbon source	PHA composition	Water contact angle [°]	Surface energy [J/cm^2^]
Acetic acid	P (99.2% HB-co-0.8% HV)	29.5 ± 5.4	64.0
Propionic acid	P (93.6% HB-co-6.4% HV)	nd	nd
Butyric acid	P (99.4% HB-co-0.6% HV)	103.6 ± 0.8	20.4
Valeric acid	P (17.5% HB-co-82.5% HV)	68.2 ± 1.3	42.2
Caproic acid	P (98.8% HB-co-1.2% HV)	78.5 ± 1.3	35.9
5% CAs—rich stream	P (98.5% HB-co-1.5% HV)	81.4 ± 0.4	34.1
10% CAs—rich stream	P (98.8% HB-co-1.2% HV)	71.3 ± 1.1	40.3
15% CAs—rich stream	P (98.3% HB-co-1.7% HV)	98.4 ± 1.1	26.6
20% CAs—rich stream	P (98.5% HB-co-1.5% HV)	64.9 ± 1.7	44.2
25% CAs—rich stream	P (98.4% HB-co-1.6% HV)	85.5 ± 0.8	31.5
30% CAs—rich stream	P (98.1% HB-co-1.9% HV)	65.5 ± 2.4	43.9

nd, not determined; HB, hydroxybutyrate; HV, hydroxyvalerate.

## Conclusion

Inexpensive carbon sources are essential to make the PHA production process economically feasible. Our results indicated that *P. homiensis* has the potential to convert CAs into PHAs which have not been reported before. As the use of pure CAs would be costly, we propose the novel idea of utilizing CAs-rich stream derived from acidogenic mixed culture fermentation of agro-industrial waste without necessity for substrate pretreatment for production of P(3HB-co-3HV) copolymer. The structure of the copolymer was analytically verified and we found that the extracted biopolymers have properties that are beneficial for industrial applications. The obtained results proved that all copolymers produced by *P. homiensis* possessed good thermal stability. However, the P(3HB-co-3HV) extracted from the cultivations supplemented with CAs-rich stream had the highest thermal resistance. These polymers show a relatively wide processing thermal window, since the difference between melting temperature and degradation temperature is higher compared to the polymers extracted from the cultivations with synthetic CAs. However, to maximize PHA production, it would be essential to extend the knowledge on metabolic pathways in *P. homiensis* that are engaged in the biopolymers synthesis.

## Methods

### Bacterial strain and inoculum preparation

The *Paracoccus homiensis* (DSMZ 17862) used in the study was purchased from the German Collection of Microorganisms and Cells Cultures GmbH in Leibniz Institute. Before inoculation into the Erlenmeyer flasks, *P. homiensis* cells were transferred from glycerol deep-frozen stock at − 80 °C to 250 mL Erlenmeyer flasks where the analyzed strain was cultured in Bacto Marine Broth at 28 °C with 120 rpm shaking for 16 h. The pre-culture medium contains the following components per liter: 5 g bacto peptone, 1 g bacto yeast extract, 0.1 g Fe(III) citrate, 19.45 g NaCl, 5.9 g MgCl_2_ (anhydrous), 3.24 g Na_2_SO_4_, 1.8 g CaCl_2_, 0.55 g KCl, 0.16 g NaHCO_3_, 0.08 g KBr, 34 mg SrCl_2_, 22 mg H_3_BO_3_, 4 mg Na-silicate, 2.4 mg NaF, 1.6 mg (NH_4_)NO_3_, 8 mg Na_2_HPO_4_. The pH-value of the medium was adjusted to 7.6 through the addition of an appropriate amount of 1 N NaOH or 1 N HCl. The medium components, together with water, were autoclaved at 121 °C.

### Carbon sources and culture conditions for PHA production

The carbon source in the culture medium were individual CAs (acetic acid, propionic acid, butyric acid, valeric acid and caproic acid) and CAs-rich stream from MCF of acid whey. Acid whey was collected from a crude cheese production line, upon usage, feedstock was stored at 4 °C. The CAs production was carried out in a 1 L working volume UASB bioreactor constructed from cylindrical plexiglass with recirculation ensuring biomass suspension as described before in Duber et al.^32^. The process was carried out at constant temperature of 30 °C and constant pH of 5.5 with automatic control using 2 M NaOH. The effluent for PHAs production was taken from a steady-state phase with high butyrate production and its characteristics were as follows: acetic acid (2.85 g/L), butyric acid (9.86 g/L), valeric acid (0.16 g/L), caproic acid (3.05 g/L), lactic acid (9.31 g/L), and ethanol (1.64 g/L).

The flask experiments were conducted in triplicate in 250 mL Erlenmeyer flasks containing 100 mL of Bacto Marine Broth (the composition described above) supplemented with varying concentration of individual CAs from 1 to 4 g/L and CAs-rich stream from 5 to 30% v/v. The CAs were added at the beginning of the cultivations. Fermentation shake flasks were inoculated with 5% v/v of the seed and were subsampled for PHA production. The cultivation with Bacto Marine Broth was treated as a control. All experiments were conducted in a rotary shaker at 110 rpm and the bacteria were incubated at 28 °C for 48 h. The cells were harvested to determine the optical density value, cell concentration, PHAs content and physico-chemical analysis of the purified biopolymers.

### Analytical procedures

Cell growth was monitored by measuring absorbance at 600 nm (OD_600_) using a spectrophotometer (Eppendorf, Germany). To obtain more accurate information on the growth of the test strain, the maximum specific growth rate (μ_max_) was determined as an equation for the linear trend line for the exponential phase. Linearization was made in which the slope of the linear regression gives μ_max_ in 1/h. To estimate cell dry mass (CDM), culture samples were harvested at 24 and 48 h and centrifuged at 9000 × *g* for 10 min. Next, the supernatant was removed and the biomass pellet was lyophilized for 24 h using Lyovac GT2 System (SRK Systemtechnik GmbH, Riedstadt, Germany). PHAs were extracted from lyophilized cells by shaking in hot chloroform at 50 °C for 3 h. Then, the mixture was filtered through No. 1 Whatman filter paper to remove cellular debris. Biopolymers dissolved in chloroform were purified by precipitation with 70% cold methanol and then allowed to evaporate at room temperature. The biopolymer content (% of CDM) was defined as the percentage of the ratio of PHAs concentration to total cell concentration.

### PHAs analysis

The monomeric composition of produced PHAs was determined by gas chromatography coupled with mass spectrometry (GC–MS QP2010 PLUS, Shimadzu, Japan) according to the method described by Możejko-Ciesielska and Pokój^33^. Firstly, the analyzed samples were suspended in mixture: chloroform–methanol-sulfuric acid (100/97/3, v/v/v) and methylation was done by heating the vials at 100 °C for 20 h. Then, sulfuric acid neutralize with Na_2_CO_3_ addition and obtained mixture was dried with anhydrous Na_2_SO_4_. After filtration methyl esters were analyzed with the use of a BPX70 (25 m × 0.22 mm × 0.25 mm) capillary column (SGE Analytical Science, Victoria, Australia) with helium as a carrier gas at a flow rate of 1.38 mL/min. The column was uniformly heated from 80 to 240 °C at the rate of 10 °C/min. The interface and ion source temperatures of GC–MS was set at 240 °C, and the electron energy was set at 70 eV. The total ion current (TIC) mode was used in 45–500 m*/z* range. Known quantities of pure 3-hydroxyacids standards (Larodan, Sweden) were used in quantitative analysis.

Infrared spectroscopy studies were performed using a FTIR Nicolet iS10 spectrophotometer (Thermo Scientific, USA). Fourier transform infrared spectroscopic spectra (FTIR) were obtained by attenuated total reflection (ATR-FTIR). Each analyzed spectrum was the average of 16 measurements recorded in the wave number range from 4000 to 650 cm^−1^. UV–Vis spectra were recorded on a spectrometer Evolution 220 (Thermo Scientific, USA) equipped with an integrating sphere. Each analyzed spectrum was the average of 16 measurements recorded in the wavelength from 200 to 1100 nm.

Differential scanning calorimetry (DSC) tests were carried out in a nitrogen atmosphere using a Q200 scanning calorimeter (TA Instruments, USA). Samples of approximately 1 mg were tested in the temperature range from − 30 to 210 °C. Due to the nature of the samples and the need to use several layers of material in one measuring crucible, the samples were heated to 210 °C in the initial stage of the test and kept at this temperature for 1 min. The sample was then cooled to − 30 °C and reheated to 210 °C. In both cases, the temperature change rate was 10 ºC/min. From the cooling and second heating curves, glass transition temperature (T_g_), crystallization temperature (T_c_), change in enthalpy of the crystallization process (ΔH_c_), cold crystallization temperature (T_cc_), change in enthalpy of the cold crystallization process (ΔH_cc_), melting point (T_m_), change in enthalpy melting process (ΔH_m_) and the degree of crystallinity (X_c_) were determined. The X_c_ values were calculated from the formula:

$${X}_{c}=\frac{{\Delta H}_{m}-{\Delta H}_{cc}}{{\Delta H}_{m100\%}}$$

where ΔH_m100%_—change in melting enthalpy of a 100% crystalline sample—109 J/g^34^.

Thermogravimetric analysis was carried out in a nitrogen atmosphere using the Q500 thermal balance (TA Instruments, USA). Samples of approximately 1 mg were tested in the temperature range from room temperature to 600 °C with a temperature change rate of 10 °C/min. Based on thermogravimetric curves, the values of T_5%_, T_50%_ and T_95%_ corresponding to the temperature of 5, 50 and 95% loss of the initial sample mass were determined. From the differential thermogravimetric curve (DTG) (first derivative of the TG curve), the T_max_ values determining the temperature of the fastest mass loss were also determined.

The hydrophilicity of biopolymers surface was evaluated by measuring the water contact angle formed between water drops and the surface of the samples using a DSA 100 goniometer (Krüss GmbH, Germany) equipped with an automatic dosing system for measuring liquid drop was used. Measurements were carried out using water (polar liquid) and diiodomethane (dispersion liquid). A drop of measuring liquid was placed on the surface of the test sample, its volume was constantly increased and at the same time the dynamic contact angle was measured, which in this case was the character of the inflow angle. After 12 measurements of contact angles with each measuring liquid, the smallest and largest values of these angles were rejected. The arithmetic mean was calculated from the remaining 10 values. Surface energy (SE) was calculated using Neumann method. The formula for the Neumann method applied to SE was in the following form:

$$cos\theta _{L} = 2\left( {\gamma _{L} /\gamma _{S} } \right)^{{0.5}} exp\left[ { - \beta \left( {\gamma _{L} - \gamma _{S} } \right)^{{0.5}} } \right]$$

where ϒ_S_- SE of polymer sample, ϒ_L_^_^ SE of water, and β = 0.0001247 (equation parameters).

### Statistical analysis

Statistical evaluation of all data was conducted using the STATISTICA v.13.1 (StatSoft, Inc, Tulsa, OK, USA). All samples were analyzed in triplicate. The Mann–Whitney U-test was used to determine the significance of differences in PHAs yield and biomass between experimental variants. Data were reported as the median ± SD at the significance level of *P* < 0.05. Spearman's correlation analysis was carried out with a significance threshold of α = 0.05.

## Supplementary Information

Supplementary Information.

## Data Availability

All data generated or analyzed during this study are present in the paper (and its Supplementary Information files).
