# Supplementary material for: Polyhydroxyalkanoates production from short and medium chain carboxylic acids by Paracoccus homiensis
Source: Sci Rep. 2022 May 4;12:7263. doi: 10.1038/s41598-022-11114-x (PMC9068790; doi:10.1038/s41598-022-11114-x)
Supplement: Supplementary file 1 — Supplementary Information. [file 41598_2022_11114_MOESM1_ESM.pdf]

Supplementary material

Polyhydroxyalkanoates production from short and medium chain carboxylic acids by *Paracoccus homiensis*

Karolina Szacherska[a], Krzysztof Moraczewski[b], Piotr Rytlewski[b], Sylwester Czaplicki[c,] Sławomir Ciesielski[d], Piotr Oleskowicz-Popiel[e], Justyna Mozejko-Ciesielska[a]*

[a] Department of Microbiology and Mycology, Faculty of Biology and Biotechnology, University of Warmia and Mazury in Olsztyn, 10-719 Olsztyn, Poland.

[b] Institute of Materials Engineering, Kazimierz Wielki University, 85-064 Bydgoszcz, Poland.

[c] Department of Plant Food Chemistry and Processing, University of Warmia and Mazury in Olsztyn, Pl. Cieszyński 1, 10-726 Olsztyn, Poland.

[d] Department of Environmental Biotechnology, University of Warmia and Mazury in Olsztyn, 10-719 Olsztyn, Poland.

[e] Water Supply and Bioeconomy Division, Faculty of Environmental Engineering and Energy, Poznan University of Technology, 60-965 Poznan, Poland.

*****Corresponding author: Justyna Mozejko-Ciesielska, Department of Microbiology and Mycology, Faculty of Biology and Biotechnology, University of Warmia and Mazury in Olsztyn, Oczapowskiego 1A, 10-719 Olsztyn, e-mail: justyna.mozejko@uwm.edu.pl, phone: (+48) (89) 5234234

Figure S1. FTIR spectra from the analysis of P(3HB-co-3HV) extracted from *Paracoccus homiensis* grown in the medium supplemented with acetic acid (A), propionic acid (B), butyric acid (C), valeric acid (D), caproic acid (E), 5% VFAs-rich stream (F), 10% VFAs-rich stream (G), 15% VFAs-rich stream (H), 20% VFAs-rich stream (I), 25% VFAs-rich stream (J), 30% VFAs-rich stream (K)

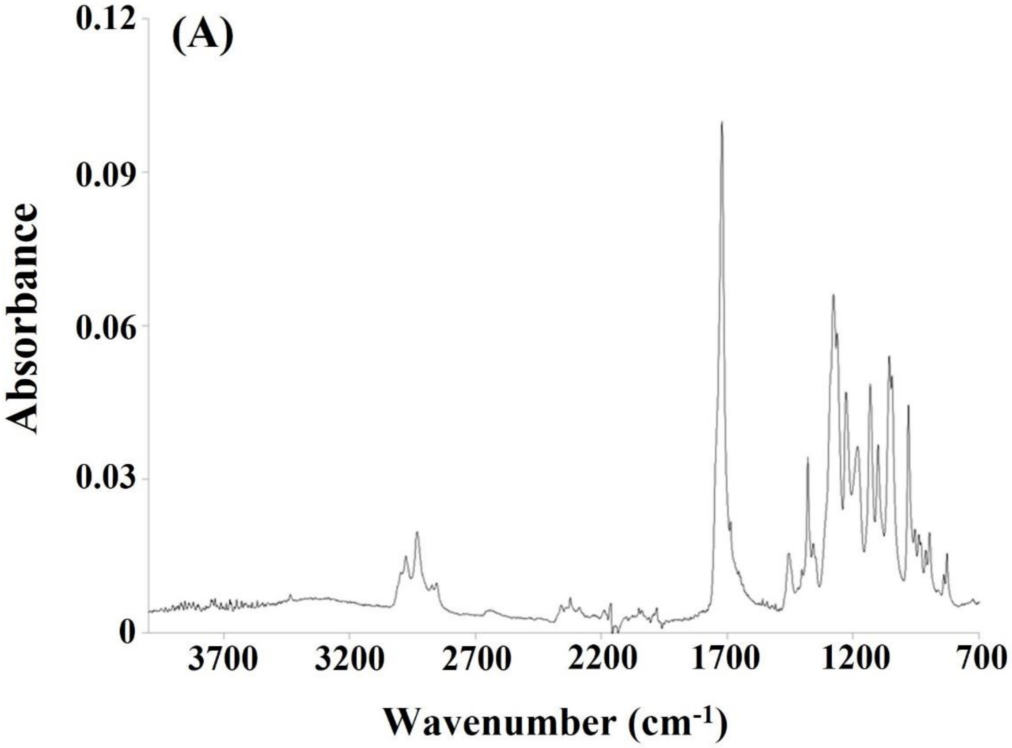

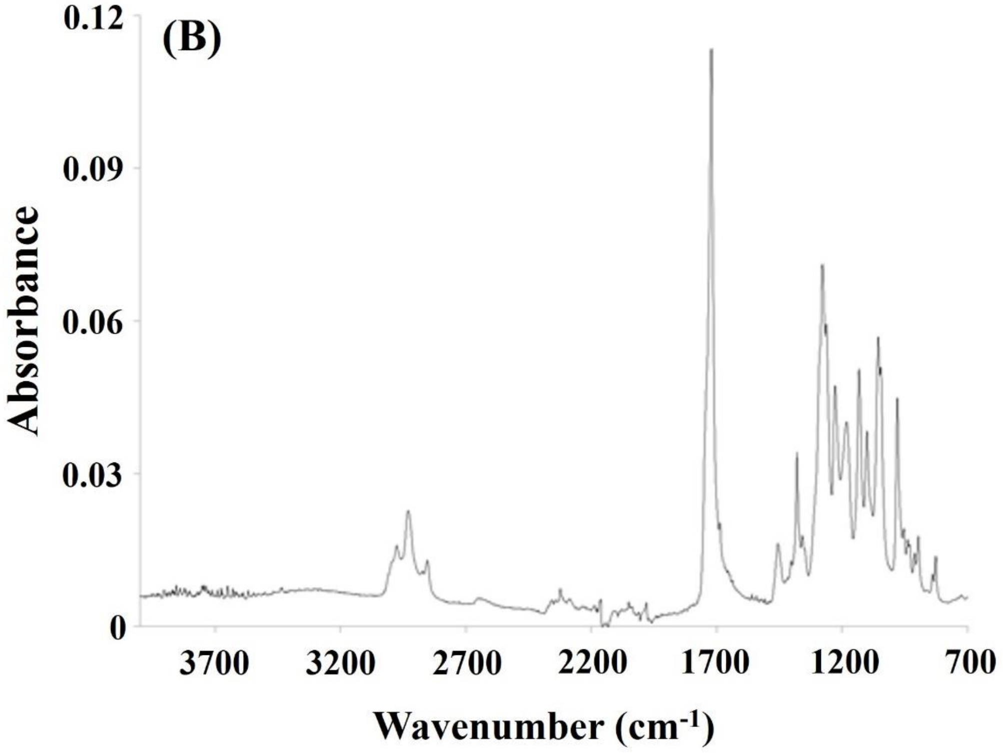

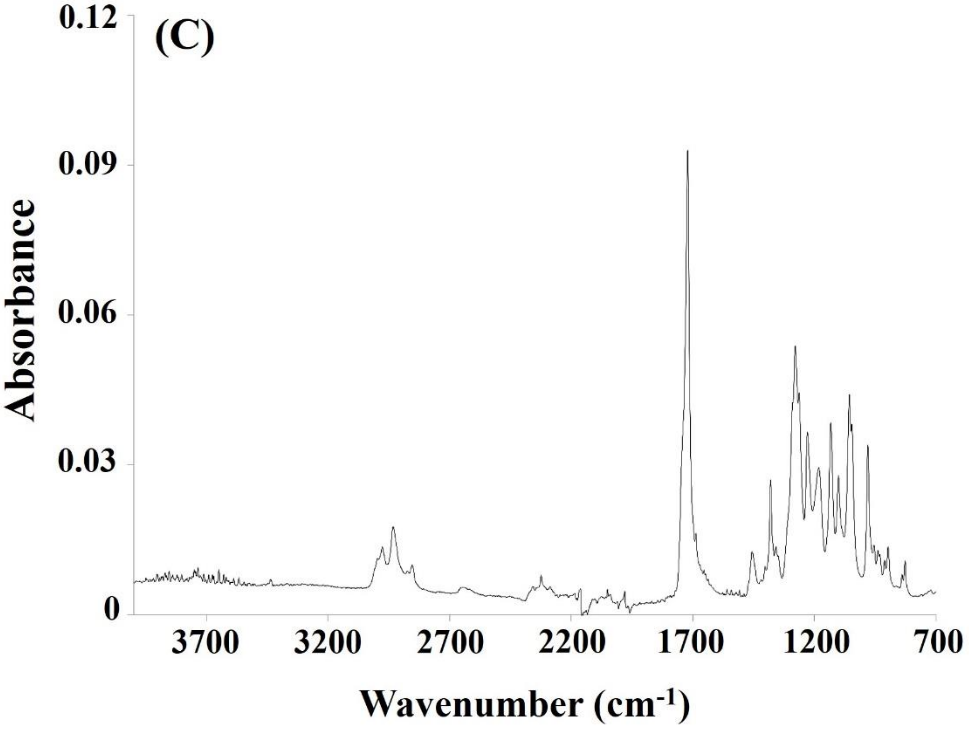

(C)
0.12
0.09
0.06
0.03
0
Absorbance
3700
3200
2700
2200
1700
1200
700
Wavenumber (cm-1)

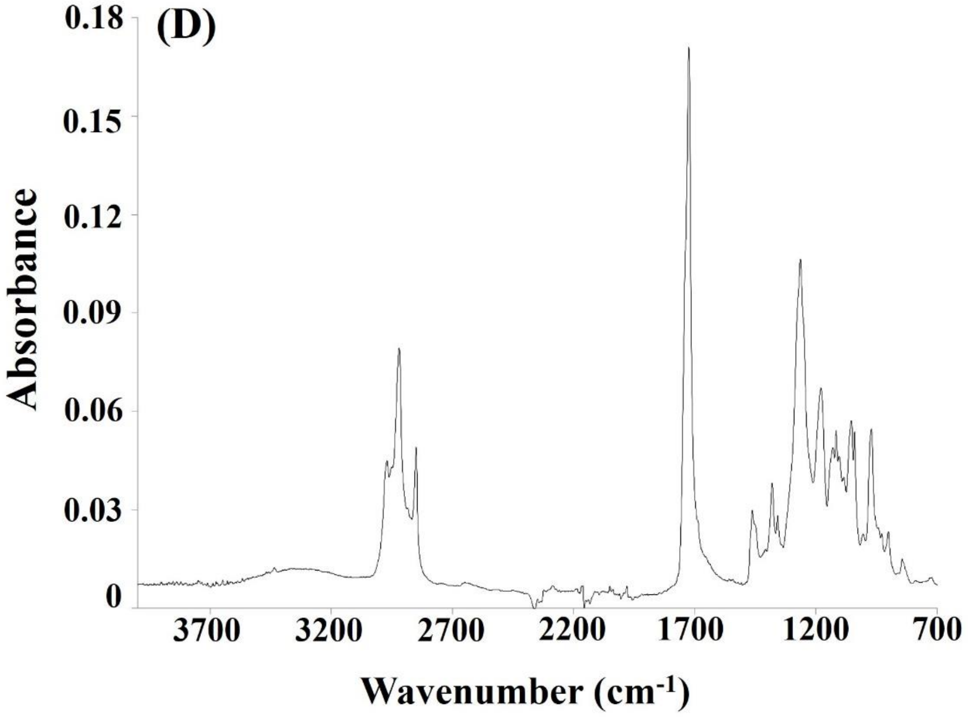

(D)
0.18
0.15
0.12
0.09
0.06
0.03
0
Absorbance
3700
3200
2700
2200
1700
1200
700
Wavenumber (cm-1)

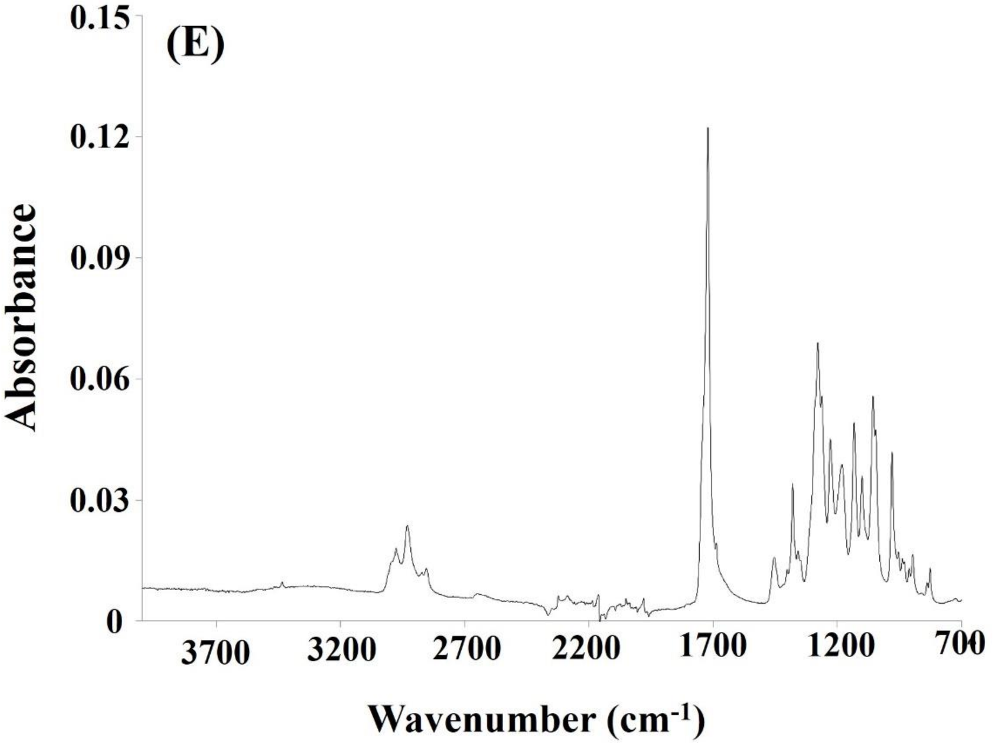
(E)
0.15
0.12
0.09
0.06
0.03
0
Absorbance
3700
3200
2700
2200
1700
1200
700
Wavenumber (cm-1)

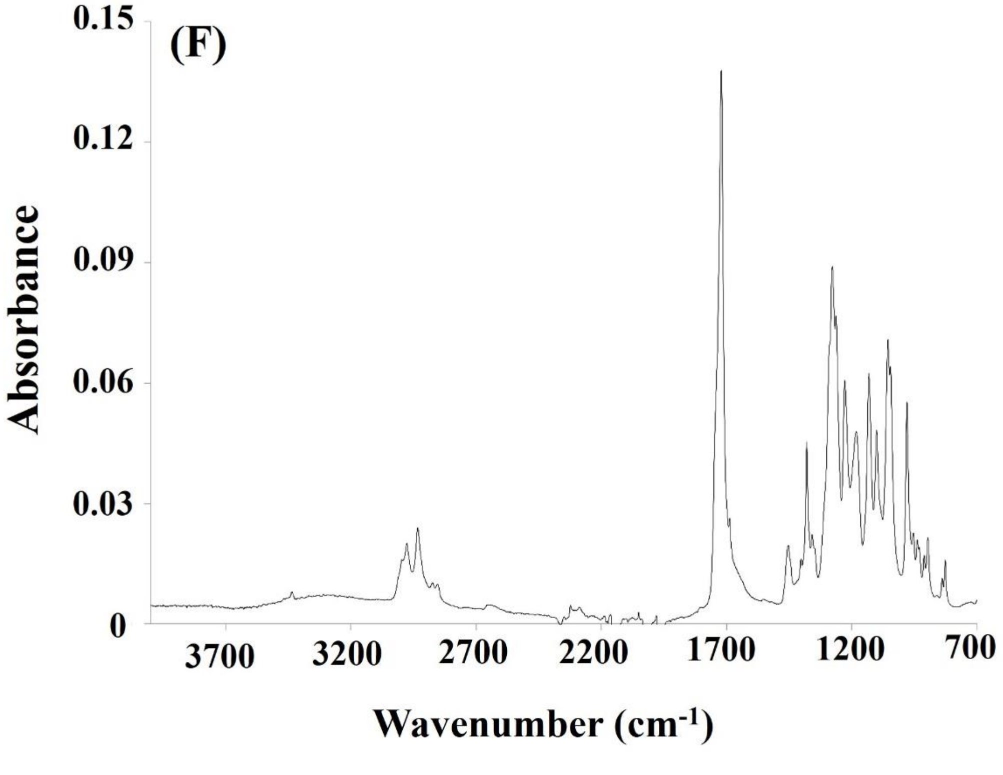
(F)
0.15
0.12
0.09
0.06
0.03
0
Absorbance
3700
3200
2700
2200
1700
1200
700
Wavenumber (cm-1)

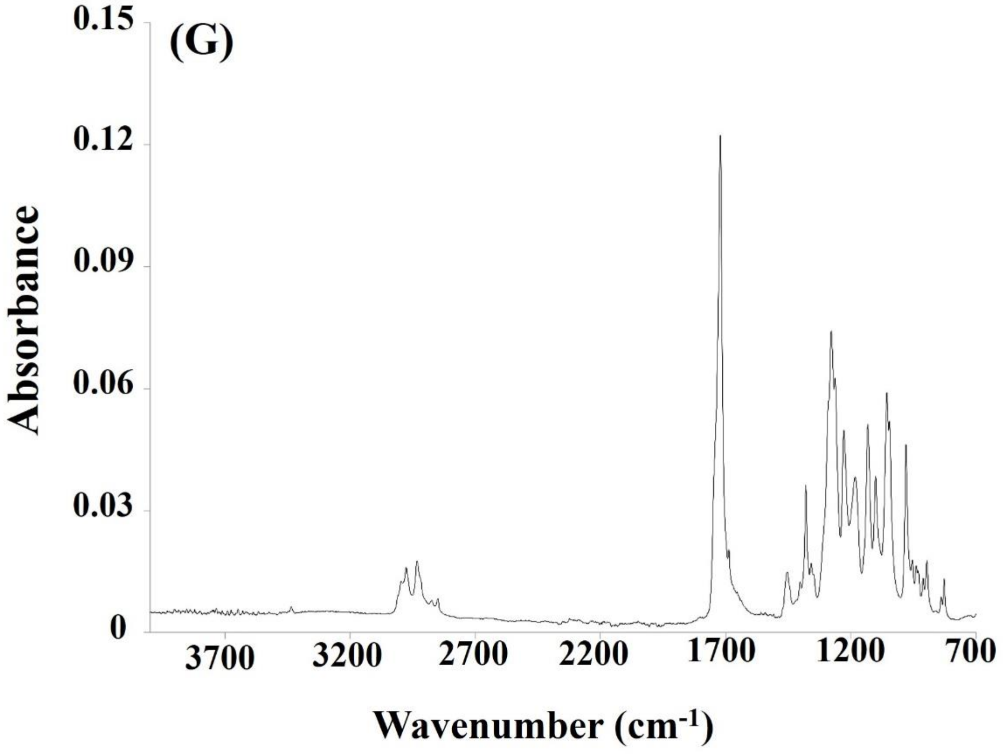
(G)
Absorbance
0.15
0.12
0.09
0.06
0.03
0
3700
3200
2700
2200
1700
1200
700
Wavenumber (cm-1)

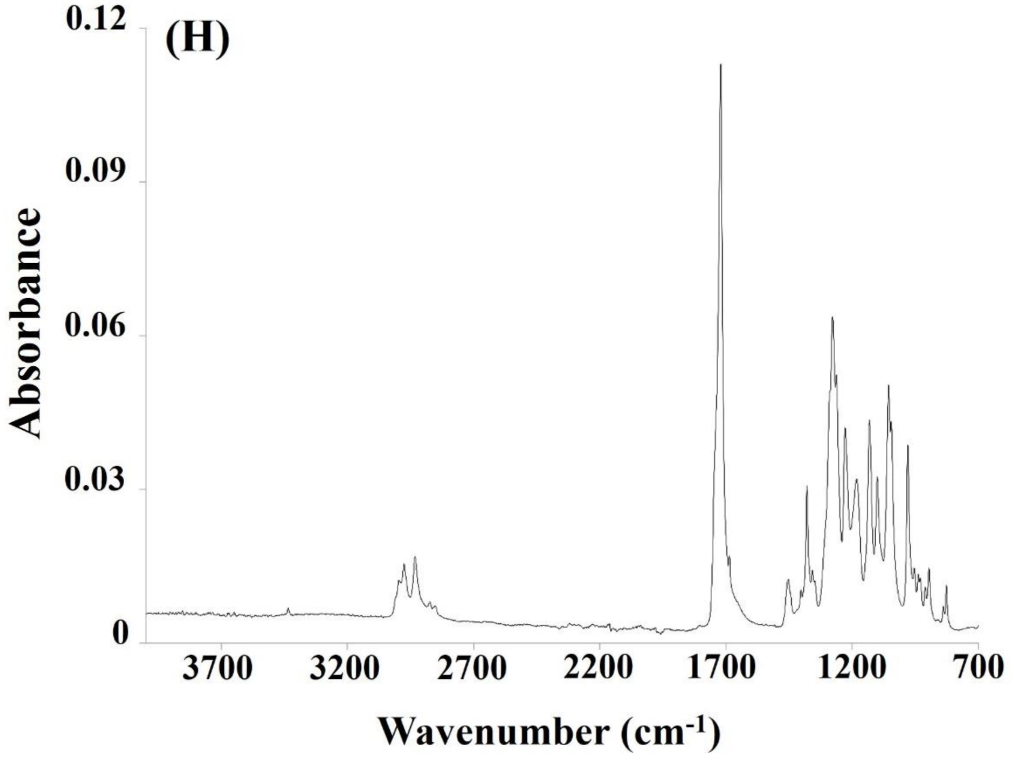
(H)
Absorbance
0.12
0.09
0.06
0.03
0
3700
3200
2700
2200
1700
1200
700
Wavenumber (cm-1)

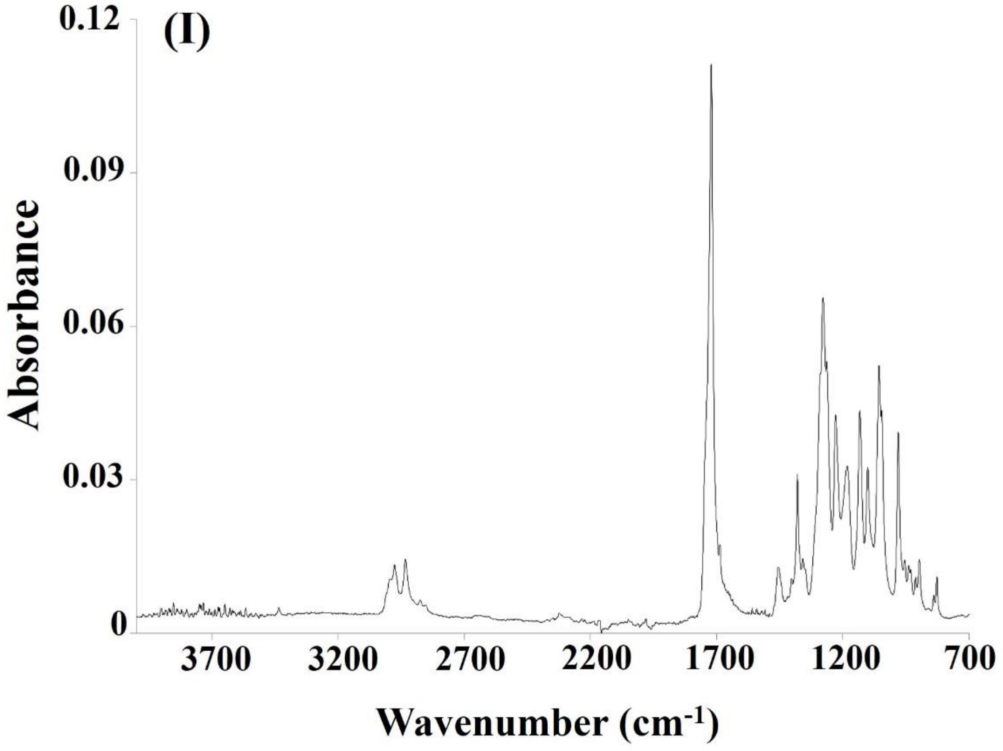
(I)
0.12
0.09
0.06
0.03
0
Absorbance
3700
3200
2700
2200
1700
1200
700
Wavenumber (cm-1)

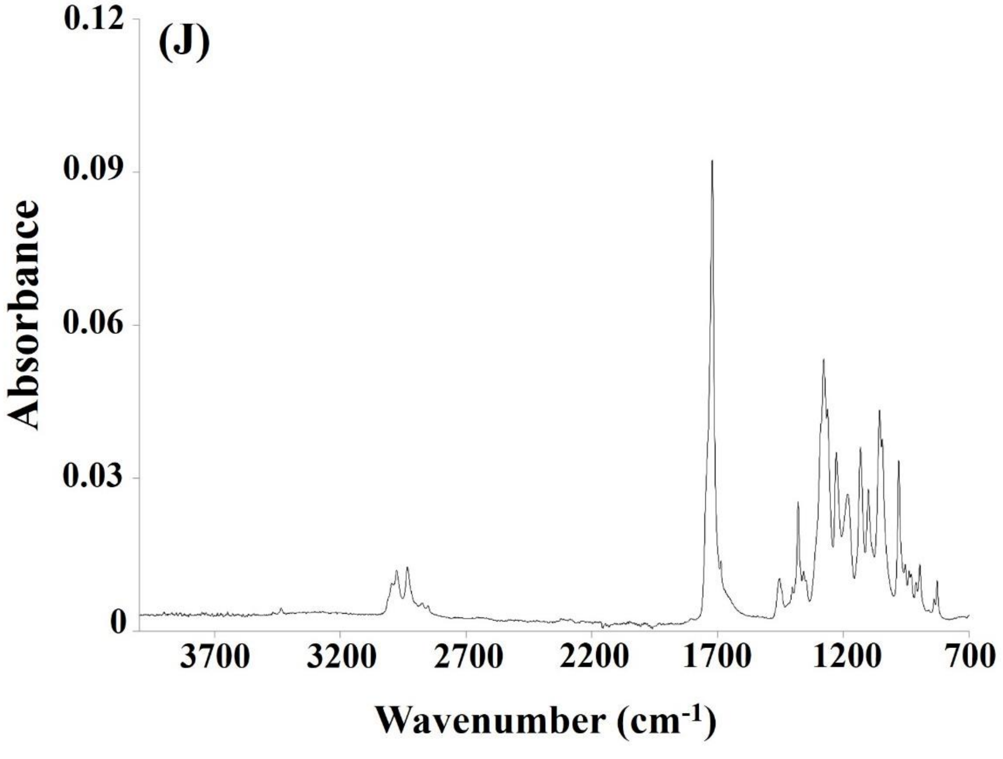
(J)
0.12
0.09
0.06
0.03
0
Absorbance
3700
3200
2700
2200
1700
1200
700
Wavenumber (cm-1)

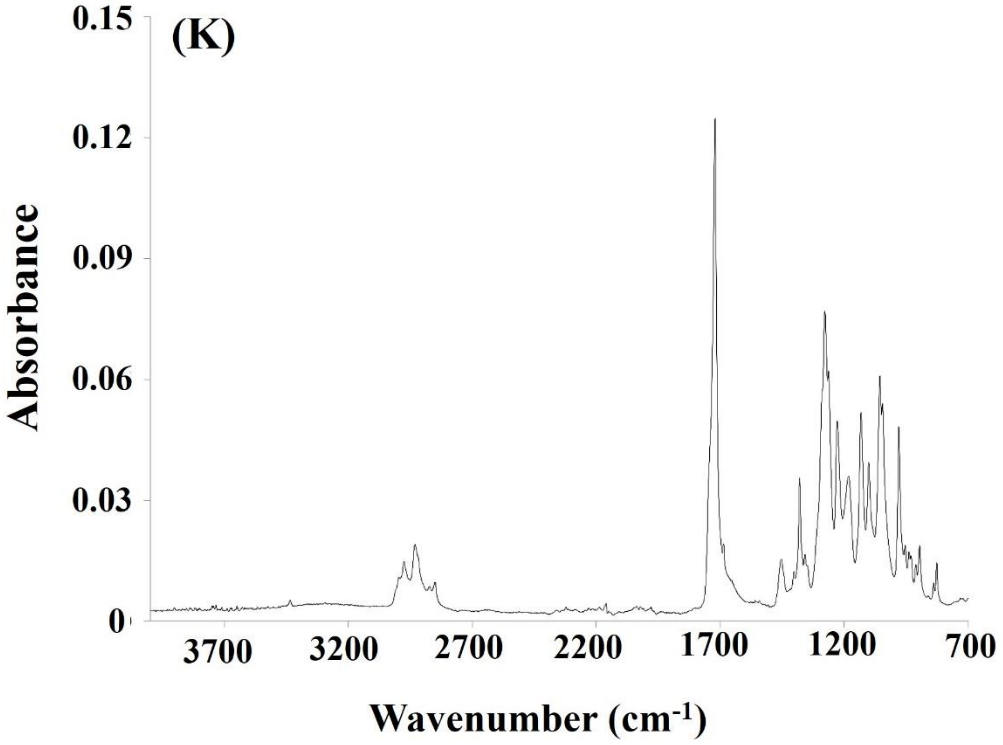

(K)
Absorbance
0.15
0.12
0.09
0.06
0.03
0
3700
3200
2700
2200
1700
1200
700
Wavenumber (cm-1)

Figure S2. UV-Vis spectra of P(3HB-co-3HV) extracted from *Paracoccus homiensis* grown in the medium supplemented with acetic acid (A), propionic acid (B), butyric acid (C), valeric acid (D), caproic acid (E), 5% VFAs-rich stream (F), 10% VFAs-rich stream (G), 15% VFAs-rich stream (H), 20% VFAs-rich stream (I), 25% VFAs-rich stream (J), 30% VFAs-rich stream (K)

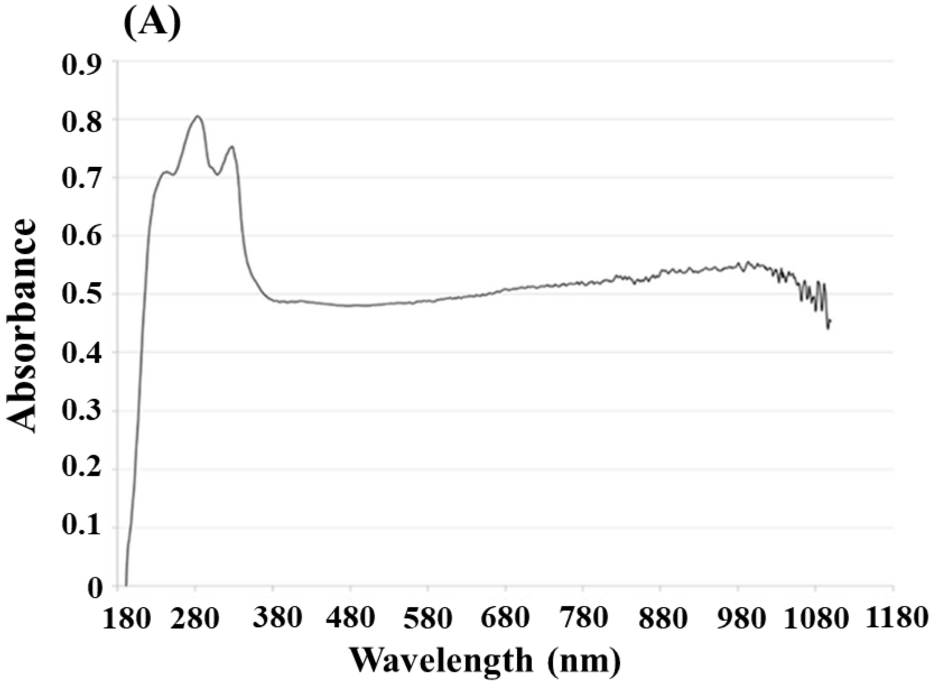

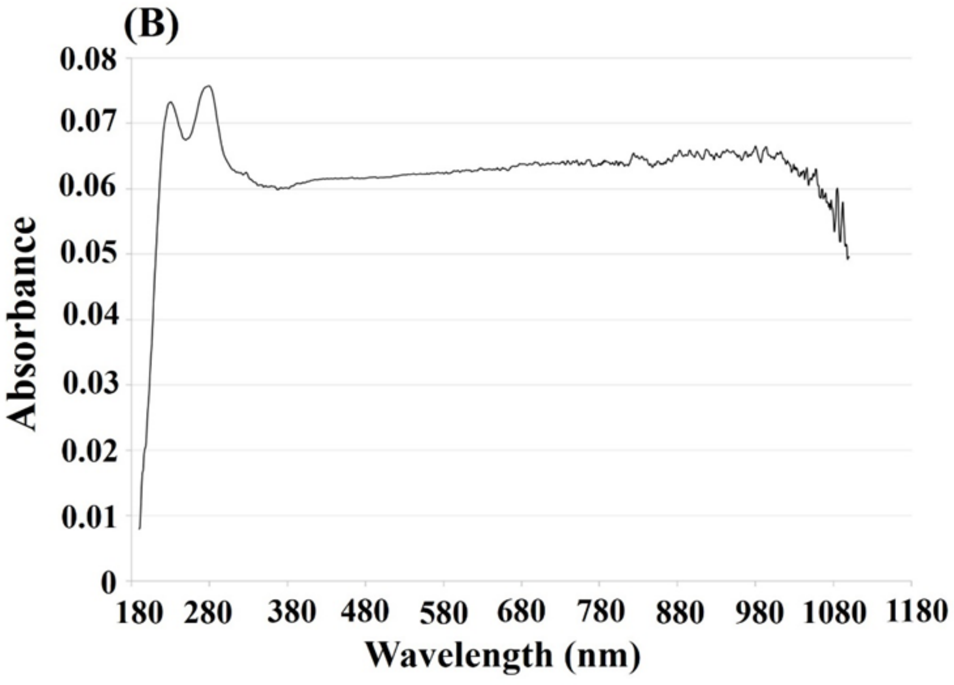

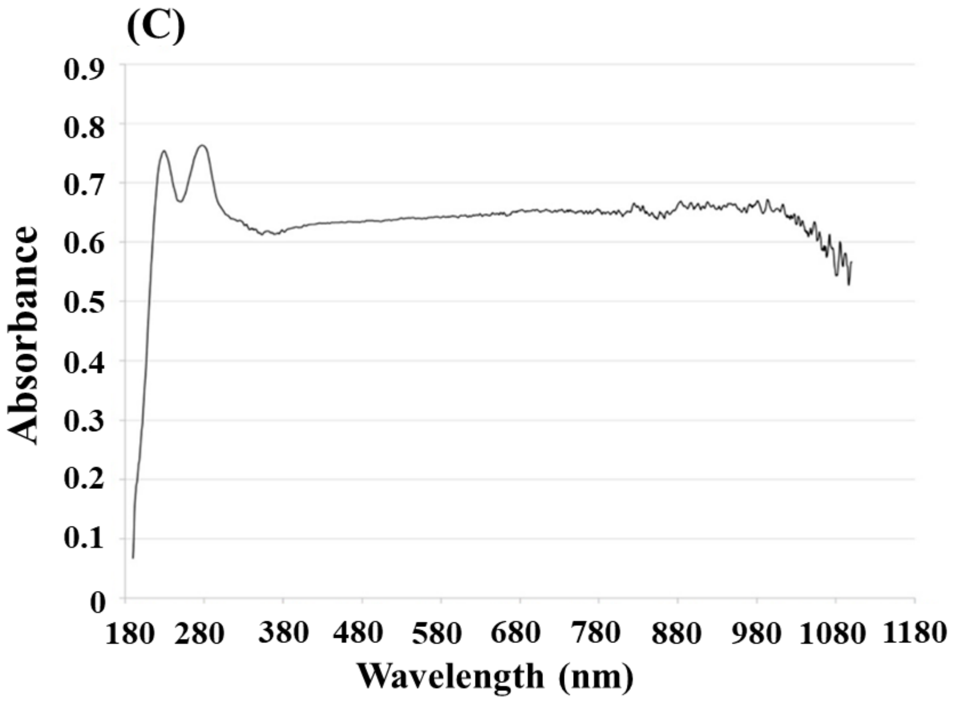
(C)
Absorbance
0.9
0.8
0.7
0.6
0.5
0.4
0.3
0.2
0.1
0
180 280 380 480 580 680 780 880 980 1080 1180
Wavelength (nm)

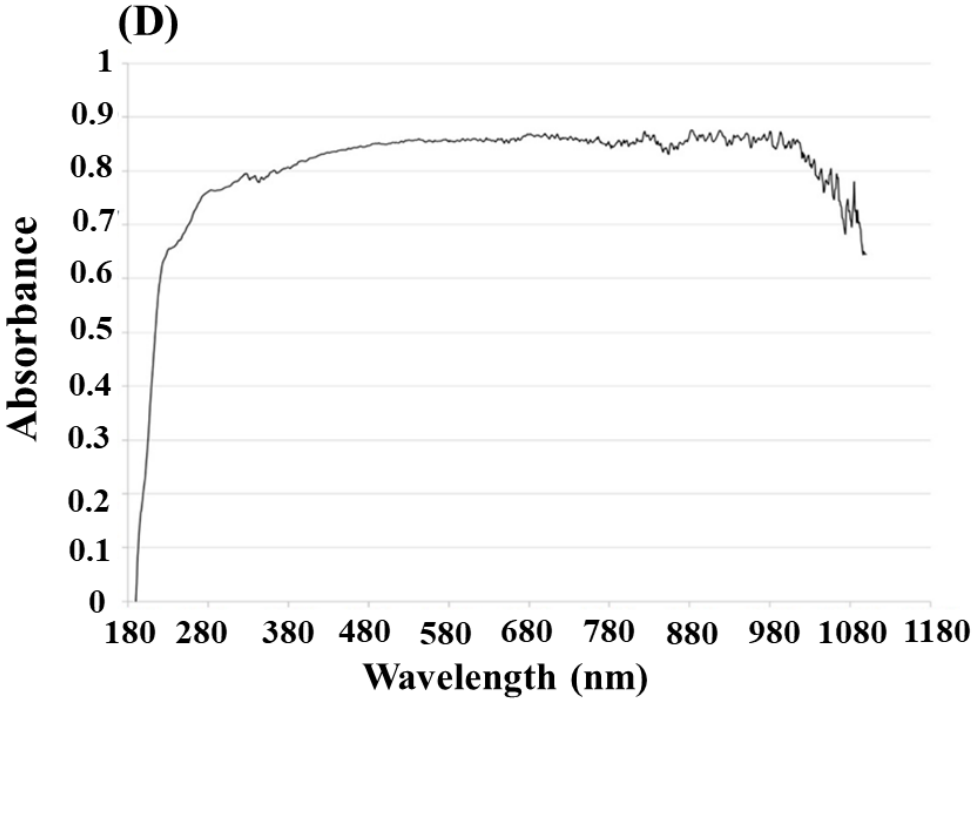
(D)
Absorbance
1
0.9
0.8
0.7
0.6
0.5
0.4
0.3
0.2
0.1
0
180 280 380 480 580 680 780 880 980 1080 1180
Wavelength (nm)

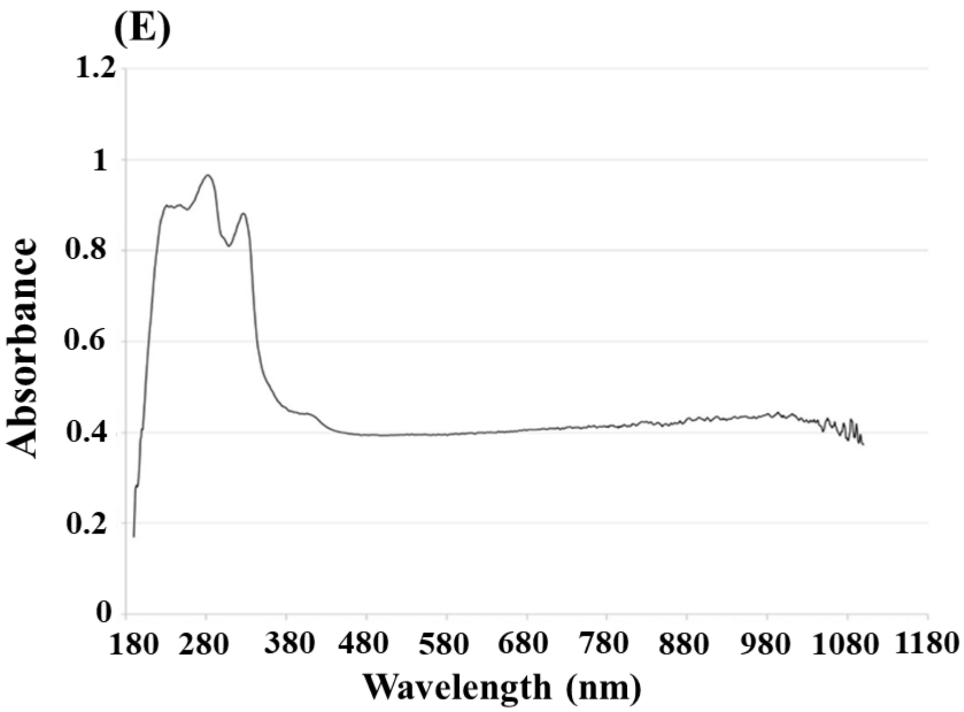

(E)
1.2
1
0.8
0.6
0.4
0.2
0
Absorbance
180 280 380 480 580 680 780 880 980 1080 1180
Wavelength (nm)

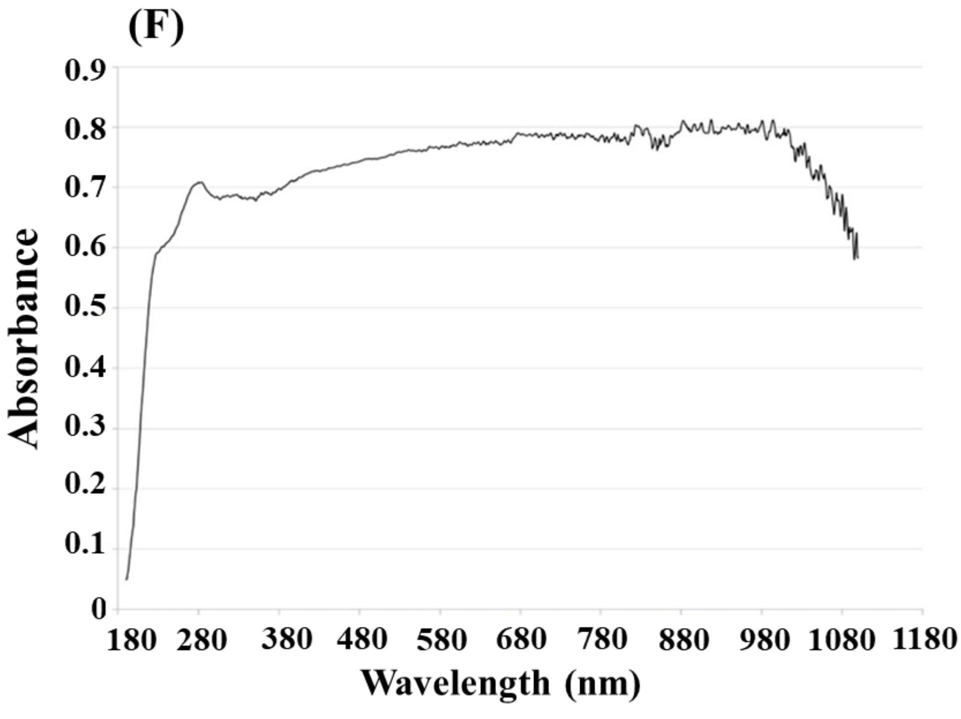

(F)
0.9
0.8
0.7
0.6
0.5
0.4
0.3
0.2
0.1
0
Absorbance
180 280 380 480 580 680 780 880 980 1080 1180
Wavelength (nm)

(G)

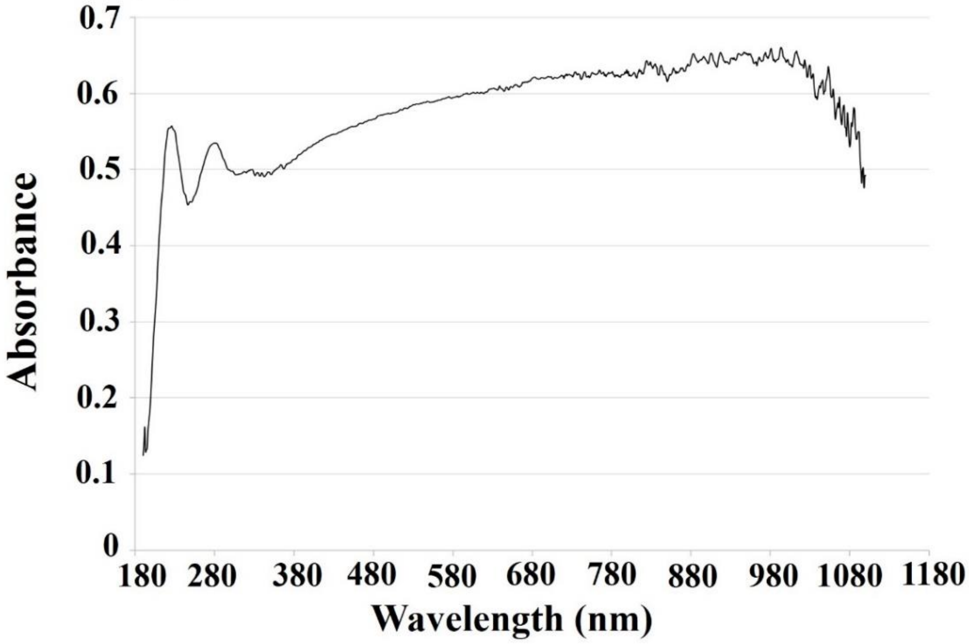
0.7
0.6
0.5
0.4
0.3
0.2
0.1
0
Absorbance
180 280 380 480 580 680 780 880 980 1080 1180
Wavelength (nm)

(H)

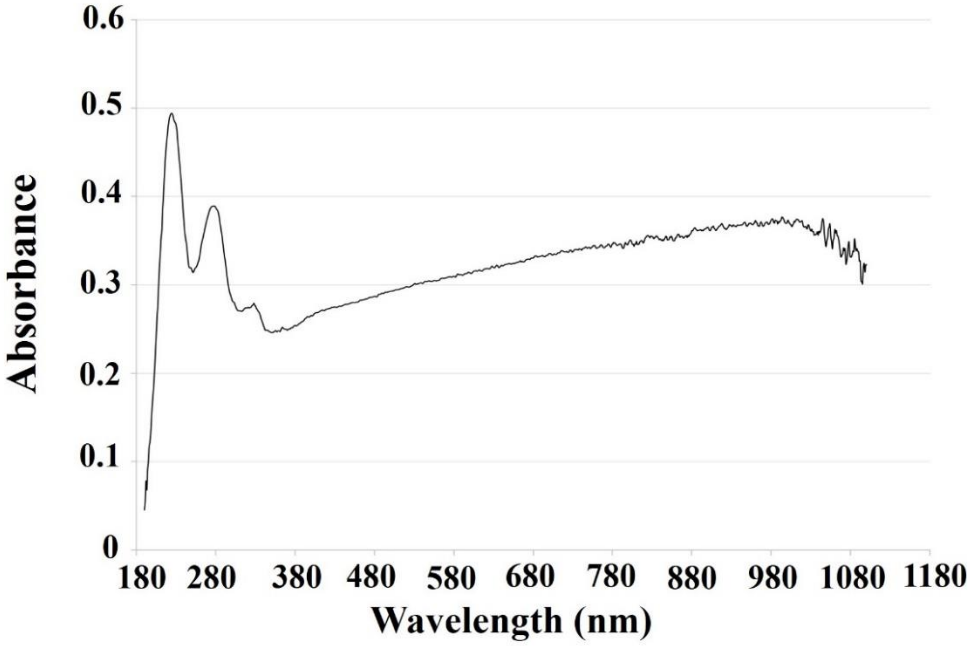
0.6
0.5
0.4
0.3
0.2
0.1
0
Absorbance
180 280 380 480 580 680 780 880 980 1080 1180
Wavelength (nm)

(I)

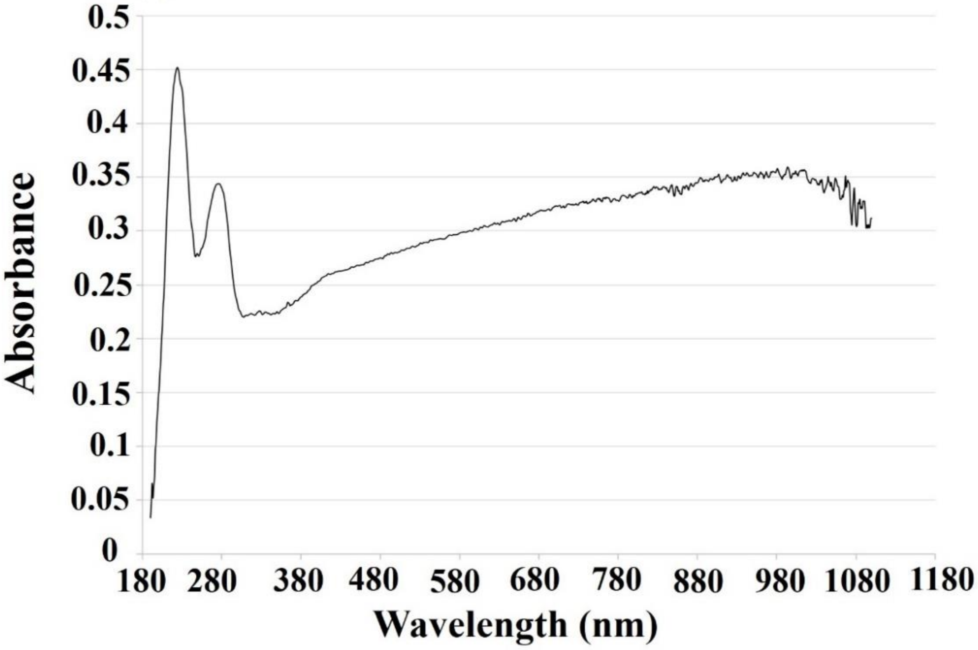

0.5
0.45
0.4
0.35
0.3
0.25
0.2
0.15
0.1
0.05
0
Absorbance
180 280 380 480 580 680 780 880 980 1080 1180
Wavelength (nm)

(J)

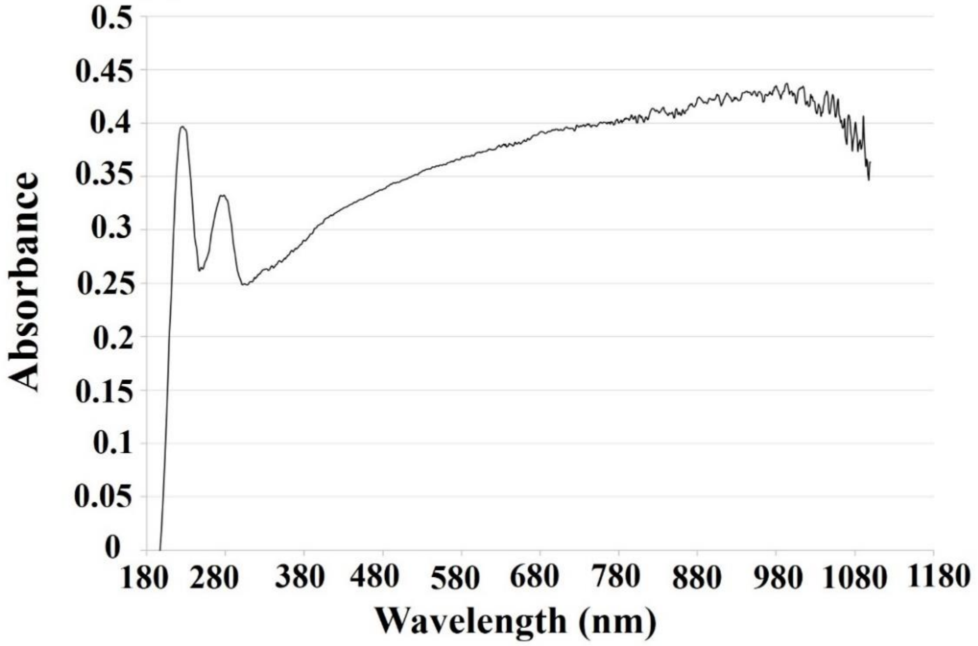

0.5
0.45
0.4
0.35
0.3
0.25
0.2
0.15
0.1
0.05
0
Absorbance
180 280 380 480 580 680 780 880 980 1080 1180
Wavelength (nm)

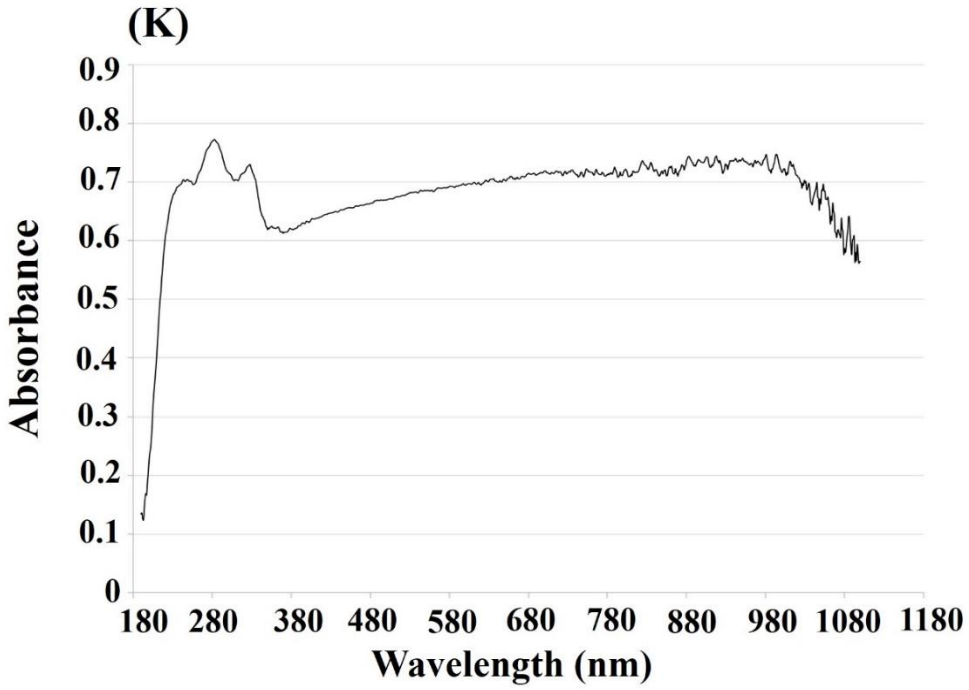

Figure S3. DSC curves of P(3HB-co-3HV) extracted from *Paracoccus homiensis* grown in the medium supplemented with propionic acid (A), butyric acid (B), 5% VFAs-rich stream (C), 10% VFAs-rich stream (D), 20% VFAs-rich stream (E), 25% VFAs-rich stream (F), 30% VFAs-rich stream (G)

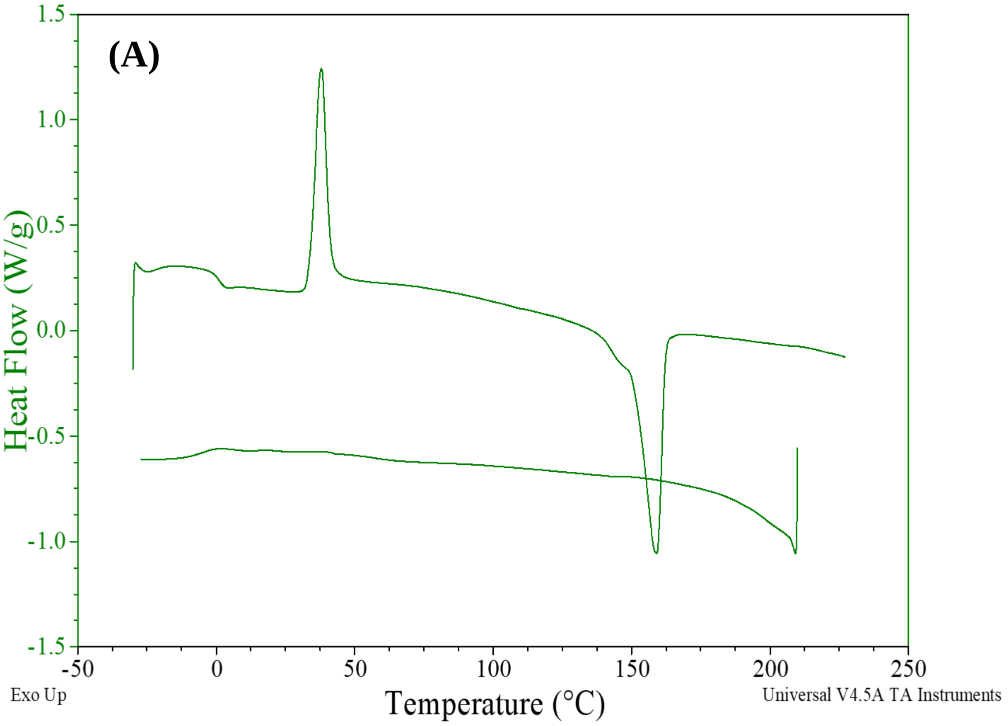

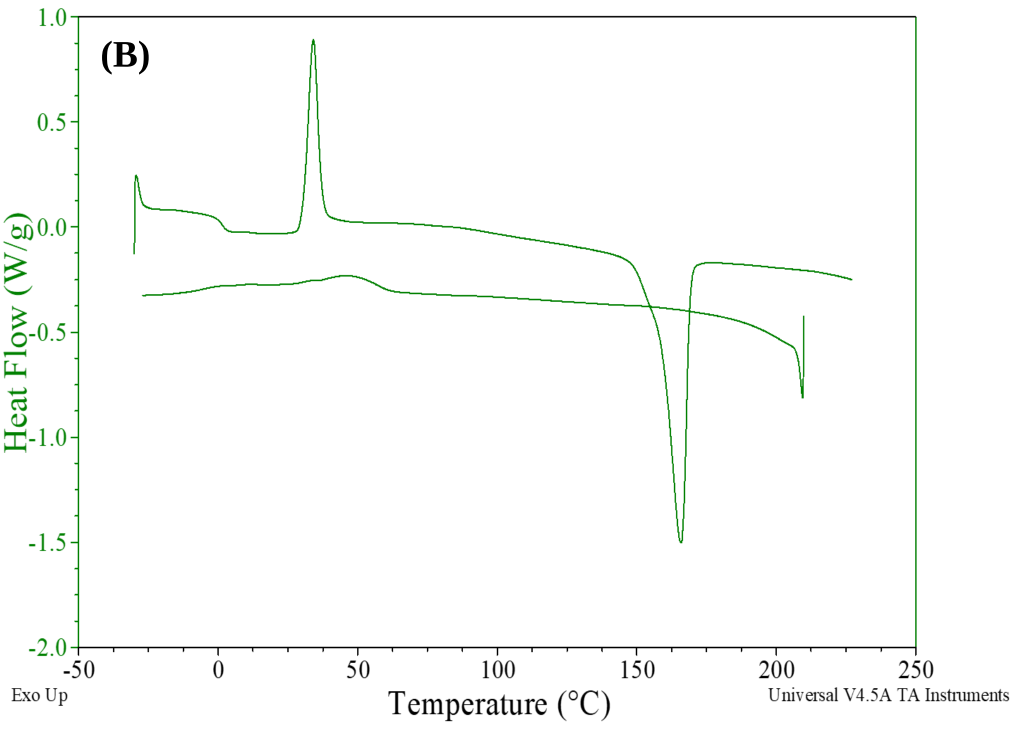
(B)
1.0
0.5
0.0
-0.5
-1.0
-1.5
-2.0
Heat Flow (W/g)
-50
0
50
100
150
200
250
Exo Up
Temperature (°C)
Universal V4.5A TA Instruments

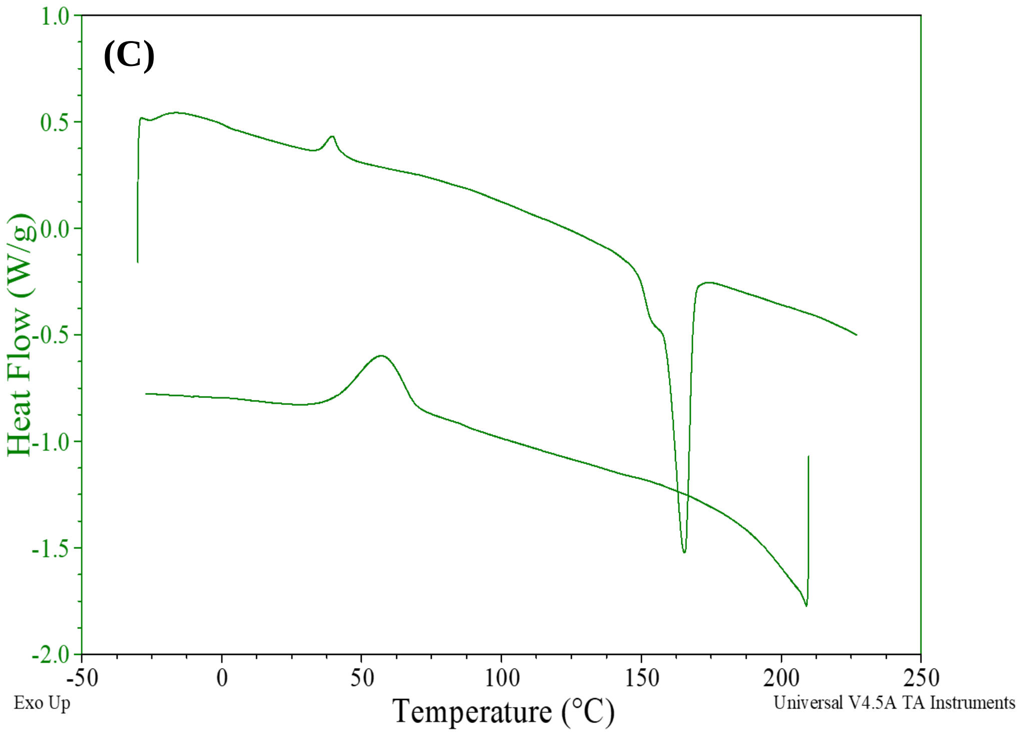
(C)
1.0
0.5
0.0
-0.5
-1.0
-1.5
-2.0
Heat Flow (W/g)
-50
0
50
100
150
200
250
Exo Up
Temperature (°C)
Universal V4.5A TA Instruments

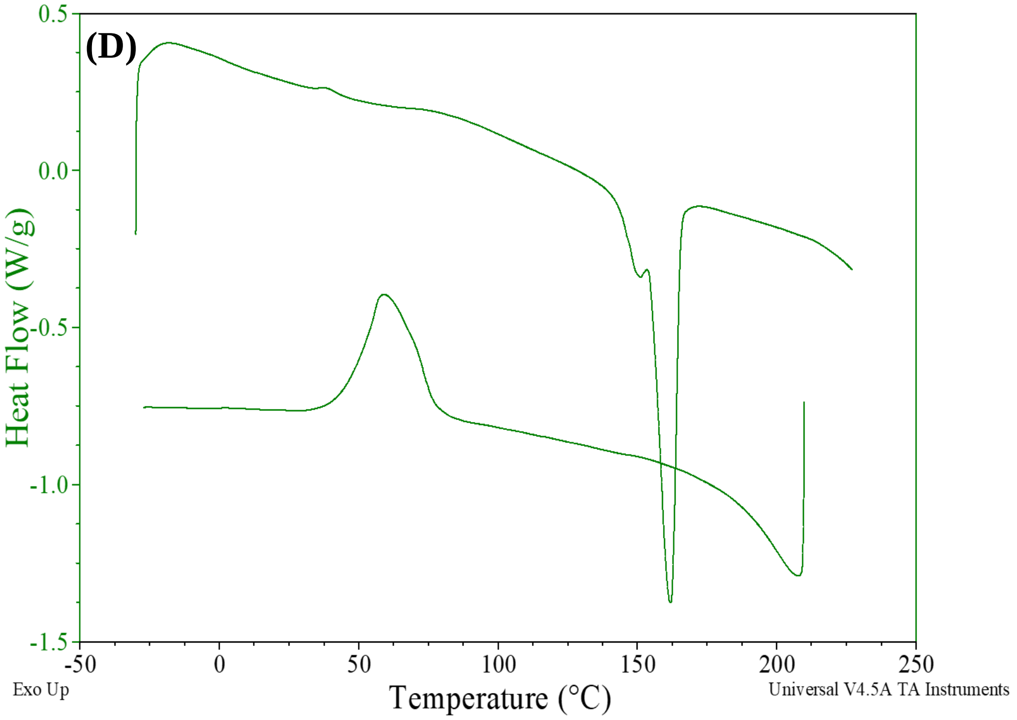

(D)
0.5
0.0
-0.5
-1.0
-1.5
Heat Flow (W/g)
-50
0
50
100
150
200
250
Exo Up
Temperature (°C)
Universal V4.5A TA Instruments

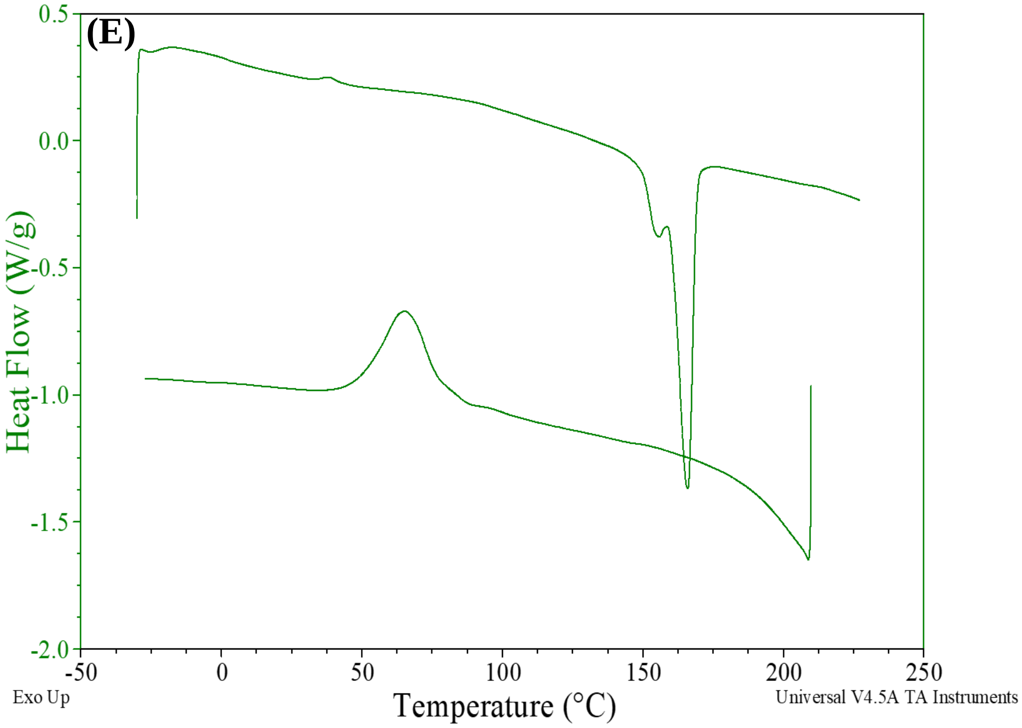

(E)
0.5
0.0
-0.5
-1.0
-1.5
-2.0
Heat Flow (W/g)
-50
0
50
100
150
200
250
Exo Up
Temperature (°C)
Universal V4.5A TA Instruments

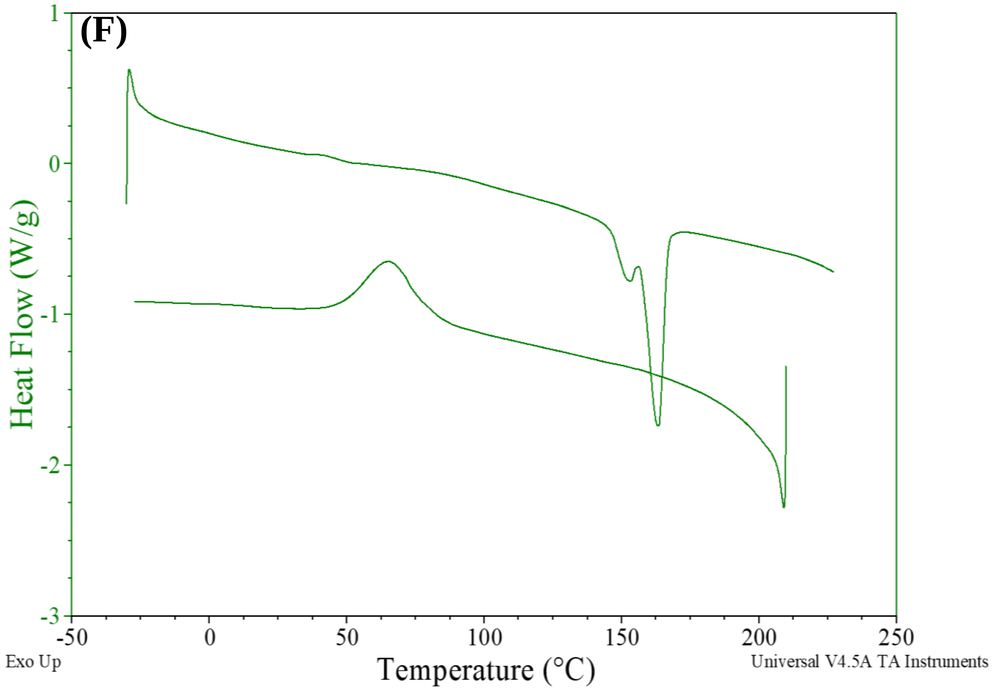
(F)
Heat Flow (W/g)
1
0
-1
-2
-3
-50
0
50
100
150
200
250
Exo Up
Temperature (°C)
Universal V4.5A TA Instruments

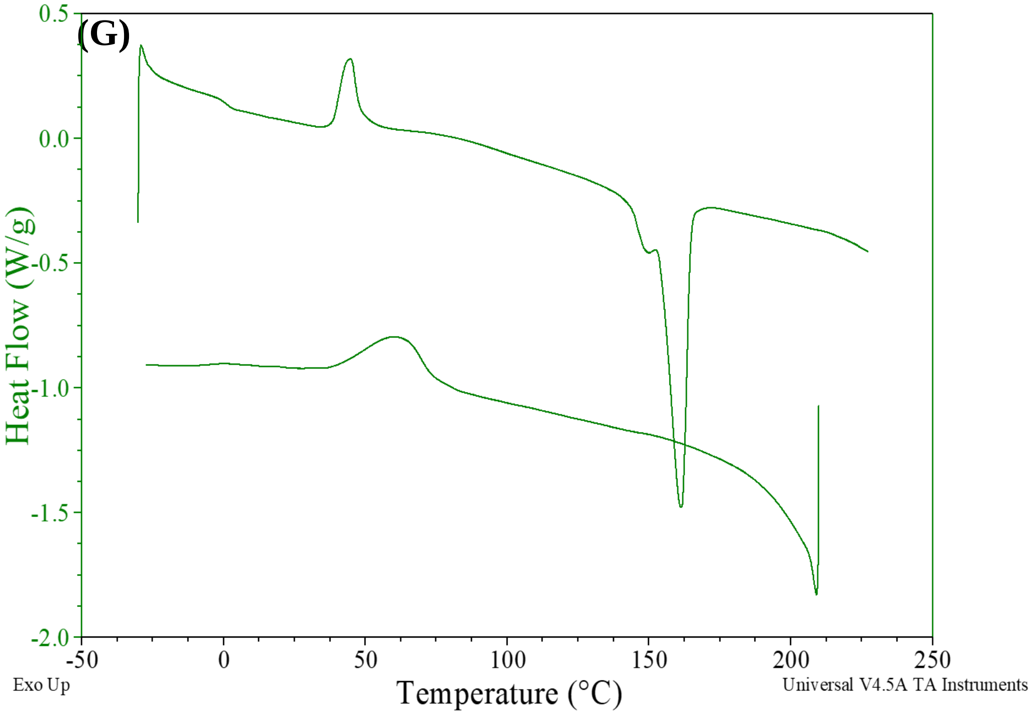
(G)
Heat Flow (W/g)
0.5
0.0
-0.5
-1.0
-1.5
-2.0
-50
0
50
100
150
200
250
Exo Up
Temperature (°C)
Universal V4.5A TA Instruments

Figure S4. TG curves of P(3HB-co-3HV) extracted from *Paracoccus homiensis* grown in the medium supplemented with propionic acid (A), butyric acid (B), 10% VFAs-rich stream (C), 15% VFAs-rich stream (D), 20% VFAs-rich stream (E), 25% VFAs-rich stream (F), 30% VFAs-rich stream (G)

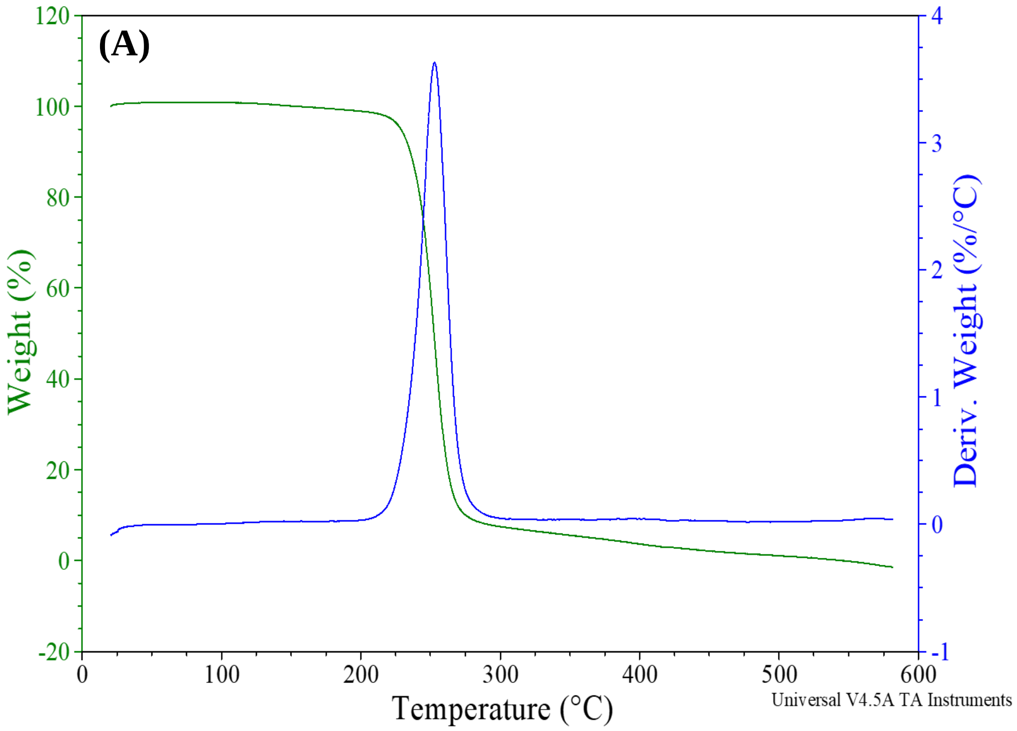

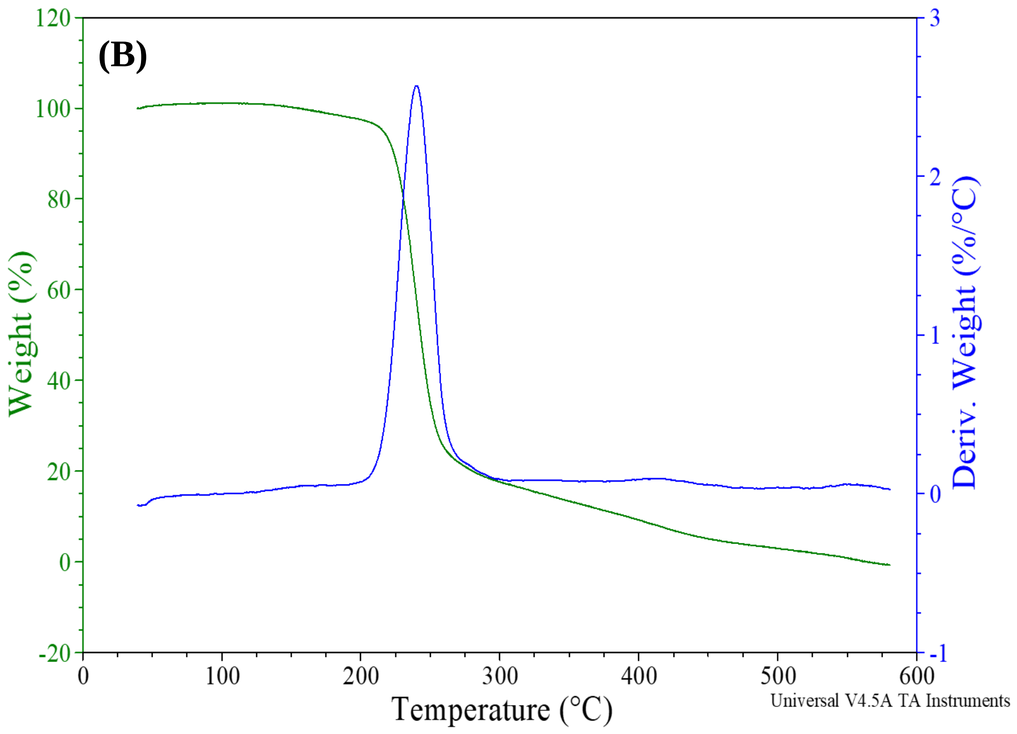

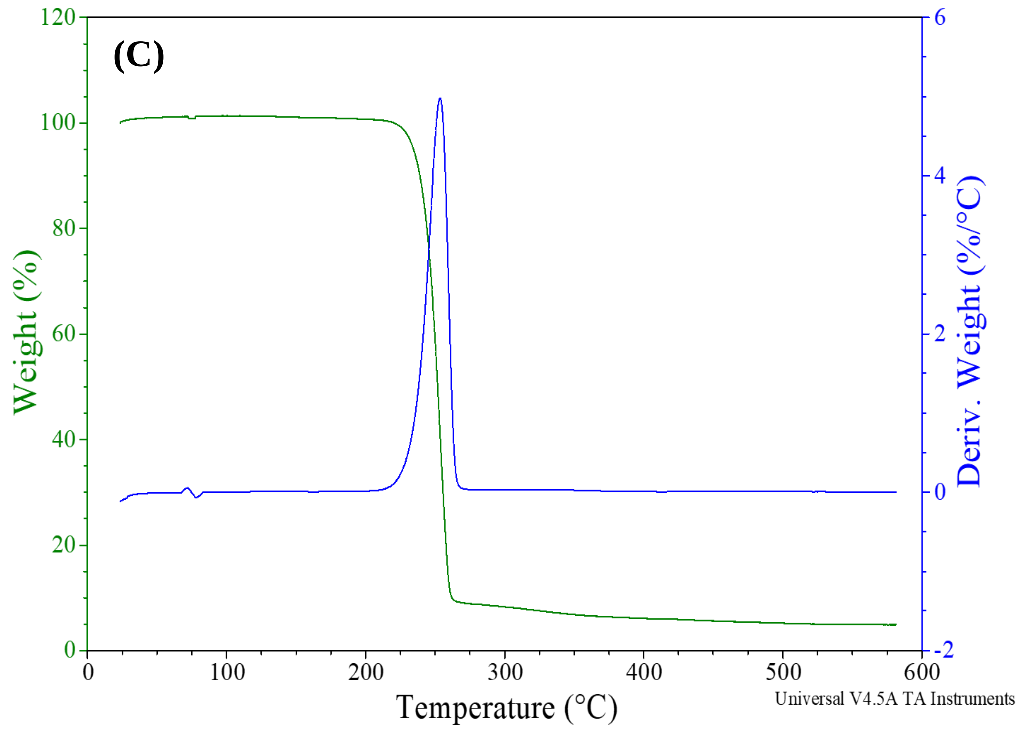
(C)
Weight (%)
Deriv. Weight (%/°C)
Temperature (°C)
0
20
40
60
80
100
120
-2
0
2
4
6
0
100
200
300
400
500
600
Universal V4.5A TA Instruments

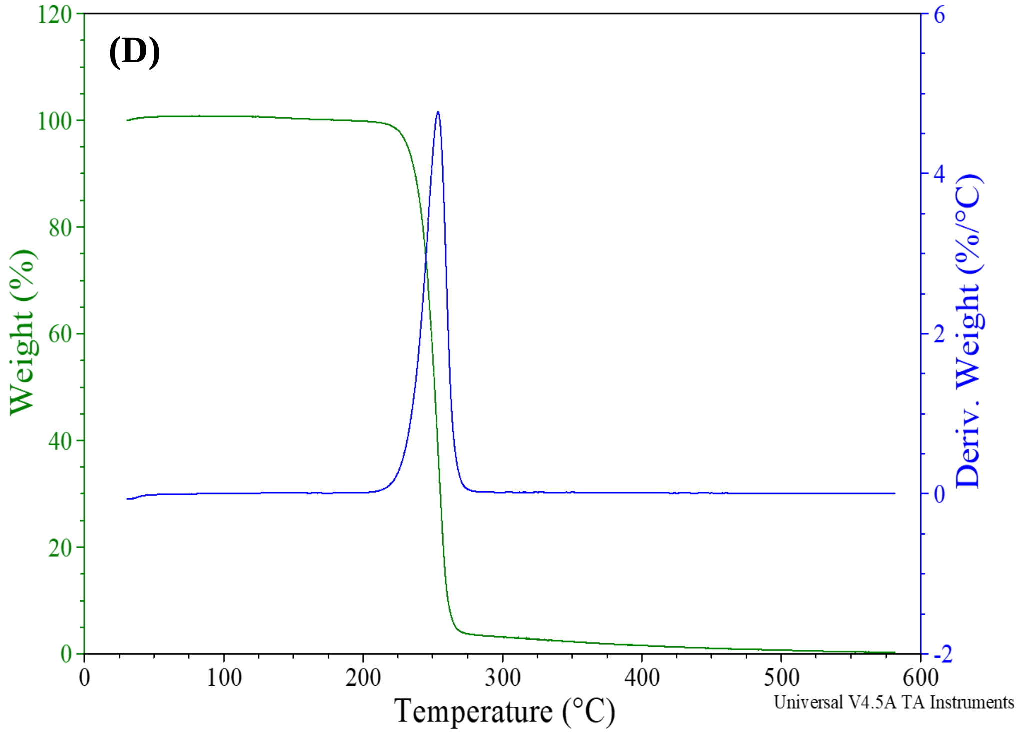
(D)
Weight (%)
Deriv. Weight (%/°C)
Temperature (°C)
0
20
40
60
80
100
120
-2
0
2
4
6
0
100
200
300
400
500
600
Universal V4.5A TA Instruments

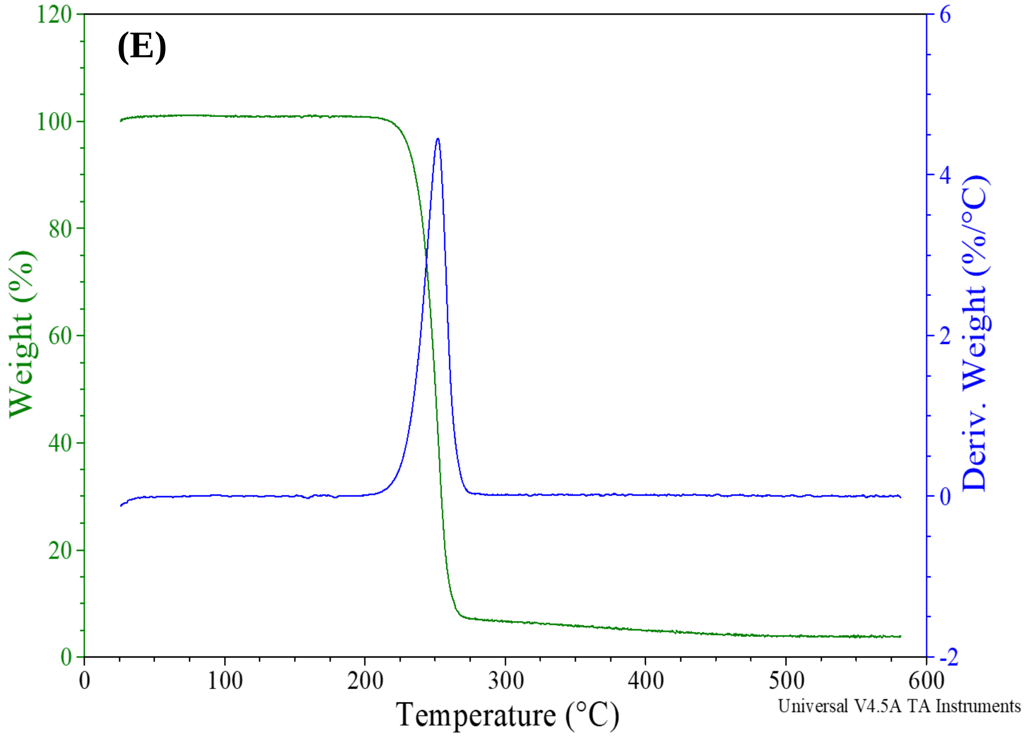

(E)
Weight (%)
Deriv. Weight (%/°C)
Temperature (°C)
Universal V4.5A TA Instruments

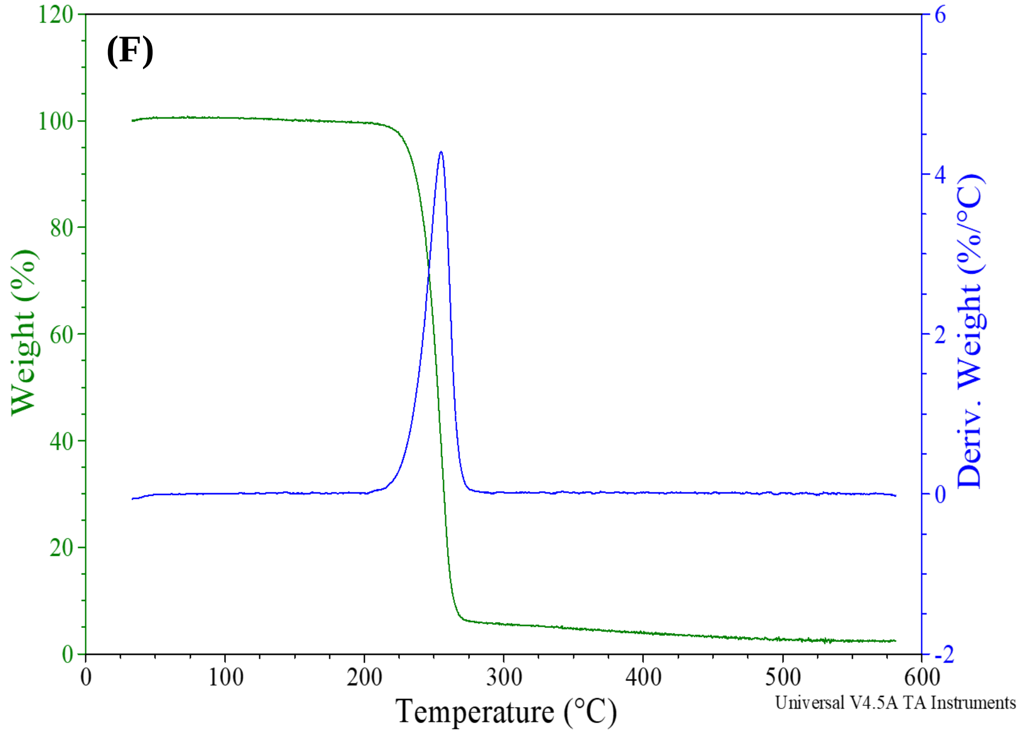

(F)
Weight (%)
Deriv. Weight (%/°C)
Temperature (°C)
Universal V4.5A TA Instruments

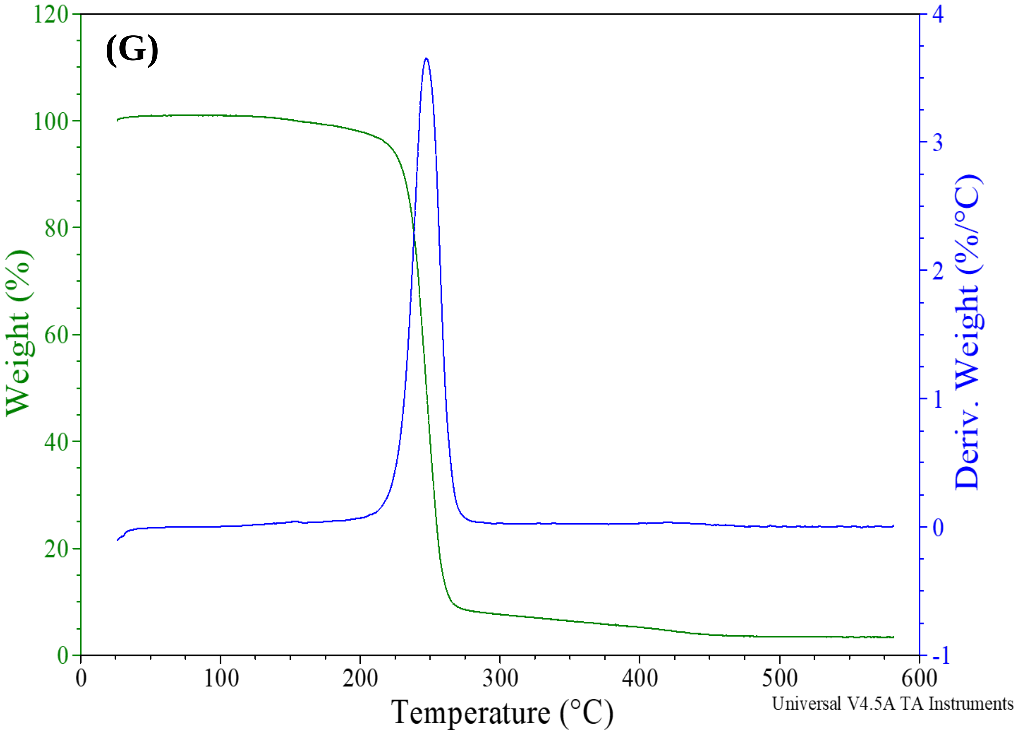
(G)
Weight (%)
Deriv. Weight (%/°C)
Temperature (°C)
Universal V4.5A TA Instruments
0
20
40
60
80
100
120
-1
0
1
2
3
4
100
200
300
400
500
600
